# Polyanionic Electrolyte Ionization Desalination Empowers Continuous Solar Evaporation Performance

**DOI:** 10.1002/adma.202410290

**Published:** 2024-12-17

**Authors:** Fengyong Lv, Jie Miao, Zhongyu Wang, Jing Hu, Daniel Orejon

**Affiliations:** ^1^ School of Urban Construction and Safety Engineering Shanghai Institute of Technology Shanghai 201418 China; ^2^ School of Energy and Power Engineering Dalian University of Technology Dalian 116024 China; ^3^ School of Perfume and Aroma Technology Shanghai Institute of Technology Shanghai 201418 China; ^4^ School of Engineering Institute for Multiscale Thermofluids The University of Edinburgh Edinburgh Scotland EH9 3FD UK; ^5^ International Institute for Carbon‐Neutral Energy Research (WPI‐I2CNER) Kyushu University 744 Motooka Nishi‐ku Fukuoka 819‐0395 Japan

**Keywords:** Donnan equilibrium effect, ionization desalination, polyanionic electrolyte functionalization, solar interfacial evaporation, superhydrophilic hierarchical metal copper foam evaporator

## Abstract

Solar evaporation contributes to sustainable and environmentally friendly production of fresh water from seawater and wastewater. However, poor salt resistance and high degree of corrosion of traditional evaporators in brine make their implementation in real applications scarce. To overcome such deficiency, a polyanionic electrolyte functionalization strategy empowering excellent uniform desalination performance over extended periods of time is exploited. This 3D superhydrophilic graphene oxide solar evaporator design ensures stable water supply by the enhanced self‐driving liquid capillarity and absorption at the evaporation interface as well as efficient vapor diffusion. Meanwhile, the polyanionic electrolyte functionalization implemented via layer‐by‐layer static deposition of polystyrene sodium sulfonate effectively regulates/minimizes the flux of salt ions by exploiting the Donnan equilibrium effect, which eventually hinders local salt crystallization during long‐term operation. Stable evaporation rates in line with the literature of up to 1.68 kg m^−2^ h^−1^ are achieved for up to 10 days in brine (15‰ salinity) and for up to 3 days in seawater from Hangzhou Bay in the East China Sea (9‰ salinity); while, maintaining evaporation efficiencies of ≈90%. This work demonstrates the excellent benefits of polyanionic electrolyte functionalization as salt resistance strategy for the development of high‐performance solar powered seawater desalination technology and others.

## Graphical abstract

Polyanionic electrolyte ionization functionalisation via layer‐by‐layer deposition as desalination strategy is implemented here to a localized thermal solar evaporator empowering continuous evaporation over extended periods of time and under real sweater from the Yellow Sea and from the East China Sea.

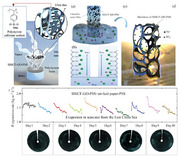

## 1 Introduction

Freshwater scarcity is intrinsic to many arid regions, which are being further impacted by the increasing demand and inefficient exploitation and utilization of freshwater resources.^[^ ^1^ ^]^ Empowered by solar radiation, seawater desalination is a promising open‐source technology enabling unlimited and stable utilization of fresh water resources without additional energy input required.^[^ ^2^ ^]^ At present, main desalination technologies, including thermal distillation and reverse osmosis, are not conducive to sustainable development because of their high energy consumption requirements with the consequent inevitable environmental pollution associated.^[^ ^3^ ^]^ Recently, interfacial solar‐driven evaporation has attracted widespread attention as a new method to generate clean water by making sole use of solar radiative energy, that is, no need for additional manmade thermal or energy intensive resources that eventually contributes to greenhouse gases.^[^ ^4^ ^]^ The photo–thermal conversion medium of the solar evaporators transforms solar energy into heat under the irradiation of the sun, heat is then transferred to the water molecules near the evaporator's interface realizing evaporation phase change, and vapor molecules are thereafter collected and condensed as clean water.^[^ ^1c^ ^]^ The effective operation of an optimized solar‐driven vapor generation system depends heavily on the excellent design of the evaporator's geometry, the selection of the photothermal materials, and the long‐term stability of the evaporation device, this latter being one of the main challenges to date for the practical implementation of interfacial solar evaporators in real applications.^[^ ^5^ ^]^

Long‐term solar‐driven seawater desalination operation typically leads to the unavoidable precipitation of salt crystals.^[^ ^6^ ^]^ Briefly, as water turns into vapor and diffuses out, the salt concentration at the evaporation interface increases, leading to crystallization at the interface or within the material pores.^[^ ^7^ ^]^ As a consequence of the salt crystallization, the absorption of the solar energy from the incident sunlight, the water transport, and the vapor diffusion paths are all weakened because of the presence of these crystals blocking the pores and paths for both the light as well as the liquid and the vapor.^[^ ^8^ ^]^ This has an irreversible impact on the evaporation device performance that eventually decays in time, leading to the reduction of freshwater production, which seriously hinders the further improvement of solar‐vapor generation performance and its practical implementation. Hence, to achieve reliable and sustainable solar evaporation, the following conditions must be met: efficient desalination of high concentration seawater, minimum energy consumption, minimum/zero environmental impact, and no degradation performance under the irradiation of the sun.^[^ ^2^, ^9^ ^]^

In recent years, a series of innovative designs has been developed to delay (salting‐out evaporation system) or prevent (salt‐free evaporation system) the shortening of the evaporator's service lifetime caused by salt accumulation.^[^ ^2a^ ^]^ On the one hand, on salting‐out or salt precipitation evaporation systems, salt crystals gradually form and accumulate at the evaporation interface as water evaporates; hence, crystals must be then removed or dissolved by the implementation of additional processes.^[^ ^10^ ^]^ Some of these processes involve a series of basic physical methods such as washing, mechanical scraping, or the implementation of hydrophobic coatings to prevent salt adhesion.^[^ ^11^ ^]^ To this end, Finnerty et al. designed a synthetic graphene oxide (GO) leaf evaporator that can easily be restored to its original state by mechanically scraping the salt crystals from its surface followed by further washing with water.^[^ ^12^ ^]^ Nonetheless, mechanical scraping may damage the actual physicochemical properties of the evaporator, requiring the shutdown of the evaporator for maintenance/replacement. Another strategy within salting‐out evaporators is the plasmonic wood solar evaporator reported by Zhu et al. that is able to diffuse the salt ions back to the bulk under no sun illumination.^[^ ^13^ ^]^ This evaporator relies on the gradual dissolution of the ion crystals absorbed within the porous structure of the evaporator back to the unsaturated water, which are then transported/diffused back into the liquid bulk driven by the concentration difference between the crystalline layer formed atop and the bulk water overnight. There are up to 144 h of operation in a simulated natural environment with 8 h of sun irradiations followed by 16 h of dark conditions cycles, which demonstrates the excellent solar vapor generation cycling performance of the ionized wood evaporator. More recently, some unique waterways or evaporator structures have been proposed so as to control local salt crystallization and its accumulation at desired sites. These evaporators rely on capillary forces driving the water flow to transfer the buildup of ions near the evaporator interface toward the bulk liquid via natural convection in a similar manner as in the case of Zhu et al.^[^ ^1c^ ^]^ To this end, the 3D acrylonitrile–styrene–acrylate (ASA) conical evaporator developed by Cao et al. is able to limit salt crystallization on the outer ring at the top of the cone, eventually avoiding the direct accumulation of salt on the evaporation surface; while, it also requires the diffusion of the ions back to the bulk water via capillary action and salt concentration gradients.^[^ ^14^ ^]^ Further, there have been other successful strategies explored. For example, Zhou et al. successfully realized an innovative self‐cleaning system enabling the self‐collection of salt driven by density difference after local salt crystallization.^[^ ^1b^ ^]^ Wu et al. proposed a 3D bionic evaporator inducing liquid film salt crystallization at the apex of the evaporator^[^ ^15^ ^]^ where crystals were subsequently self‐removed by gravity action.^[^ ^7^, ^10^ ^]^ Due to the preferential crystallization at the edge coupled to the gravity‐assisted self‐cleaning capabilities, this system could continuously purify natural seawater samples at the rate of 1.72 kg m^−2^ h^−1^ for 10 days in a closed system.^[^ ^15^ ^]^

On the other hand, salt‐free systems refer to the continuous solar evaporation without the occurrence of crystallization within the pores or at the surface of the evaporator.^[^ ^16^ ^]^ The key to fundamentally inhibit salt crystallization is to maintain low salt ion concentration at the evaporator's surface by making the salt ions to either not diffuse through the evaporator or to diffuse back to the bulk liquid/brine.^[^ ^17^ ^]^ Recently, researchers have proposed a double‐layer Janus structure able to diffuse the salt ions at evaporators interface back to the brine during the continuous pumping process.^[^ ^18^ ^]^ In order to achieve this, the light collection and water pumping paths in this specific solar evaporator are separated by a Janus membrane, which results in the nucleation of salt ions only occurring in the hydrophilic layer, which are then gradually dissolved without causing the loss of light absorption as otherwise would occur in the presence of salt crystals. More recently, a self‐contained Janus aerogel that provides sufficient water flux through the vertically arranged micro‐paths was developed by Liu et al.^[^ ^19^ ^]^ Such configuration allows the salt ions to be transferred back to the bulk solution through diffusion and advection, which are controlled by the salt concentration gradient. However, the structure of the Janus membrane needs to be thin so to avoid salt accumulation at the separation interface, which hinders high robustness as well as high evaporation efficiency.^[^ ^20^ ^]^ In addition, the longitudinal arrangement and stacking characteristics of the membranes increase the mass transfer resistance for the diffusion of water from the bulk, which leads to the reduction of the evaporation rate.

**Figure 1:**
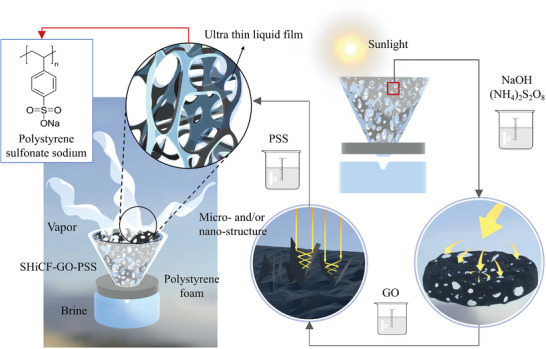
Schematics on the functionalization and working principles of the superhydrophilic copper foam graphene oxide polystyrene sodium sulfonate SHiCF‐GO‐PSS evaporator wrapped in air‐laid PSS‐paper.

Hence, to inhibit salt crystallization, it is therefore important and desirable to control the salt concentration in the water supply path instead.^[^ ^17^, ^21^ ^]^ To this end, we exploit the Donnan effect so to counteract the formation of salt crystals by limiting the transport of specific salt ions toward the evaporator's interface. Based on the Donnan effect, cations near the evaporator's inlet such as Na^+^ are immobilizez; hence, keeping a high Na^+^ chemical potential, hindering the further movement of Cl^−^ anions from the saltwater bulk toward the inlet.^[^ ^22^ ^]^ Such increase in Na^+^ cations is achieved by the introduction of additional anions at the inlet of the evaporator.^[^ ^23^ ^]^ To date, most of the solar evaporators exploiting this strategy make use of functionalized hydrogels where negative charges are fixed on the structural wall making them salt resistant.^[^ ^23^, ^24^ ^]^ To this end, Zhao et al. made use of polyelectrolyte hydrogel (free radical polymerization of acrylic acid and crosslinker *N*,*N*′‐methylenebis(acrylamide)) functionalization at the water supply inlet path, impeding the transfer of ions from the bulk brine to the evaporator; while, carbon fibers at the evaporator's top additionally provides salt resistant capabilities.^[^ ^23^ ^]^ The presence of the polyelectrolyte hydrogel induces a Donnan distribution balance at the interface between the water supply path and the bulk salt water, which greatly reduced the amount of salt ions diffused toward the evaporator's surface.^[^ ^23^ ^]^ In this work, we introduce a 3D conical superhydrophilic copper foam graphene oxide evaporator SHiCF‐GO, which had been further functionalized following strategies introduced in nanofiltration such as layer‐by‐layer static deposition of a polyanion electrolyte, that is, polystyrene sodium sulfonate (PSS), as represented in **Figure** **1**. The easy and scalable layer‐by‐layer static deposition of the polyanion electrolyte functionalization could be carried out without the need for various advanced equipment and complex chemicals or preparation procedures. Moreover, the layer‐by‐layer static deposition procedure adopted enables the use of less bulk material and chemicals; while, the copper foam skeleton provides additional robustness when compared to recently proposed hydrogels imparting the Donnan effect.^[^ ^25^ ^]^ The solar evaporator modified by the PSS polyelectrolyte provides similar liquid and vapor transport; and hence, similar evaporation rates and efficiencies as for the original not functionalized SHiCF‐GO evaporator.^[^ ^23^, ^26^ ^]^ Moreover, the maximum evaporation rate and solar absorbance reported here for our SHiCF‐GO evaporator are within the average and standard deviation when comparing it with current literature values as summarized in Table S4 and Section S17, Supporting Information. A maximum evaporation rate of 1.71 kg m^−2^ h^−1^ and a solar absorbance of 93.6% are reported for our evaporator when compared to 1.83 ± 0.62 kg m^−2^ h^−1^ and 95.2% ± 2.5% estimated from Table S4, Supporting Information. At the same time, the implementation of the PSS polyelectrolyte provides the additional necessary resistance to the ions transport; hence, avoiding salt crystallization, which eventually empowers the long‐term performance operation in brine of up to 90 h without service or maintenance when compared to the average of the long‐term operation research works introduced in Table S4, Supporting Information of 40 ± 27 h. Moreover, it also represents the longest of the long‐term operation durations for real sweater desalination reported in Table S5, Supporting Information as the work by Zhu et al. reporting up to 144 h of operation or the work of Wu et al. reporting up to 200 h of operation did only consider simulated seawater and no real seawater.^[^ ^13^, ^27^ ^]^

To sum up, the proposed SHiCF‐GO‐PSS yields evaporation rates as high as 1.68 kg m^−2^ h^−1^ in high concentration brine with a salinity of 15‰ and in real seawater from Hangzhou Bay in the East China Sea with a salinity of 9‰ over prolonged periods of time. Moreover, experiments making use of higher salinity of 35‰ and hence greater number of ions in solution yields evaporation rates ranging between 1.43 and 1.61 kg m^−2^ h^−1^. Although a daily decay is observed, the evaporator configuration proposed does recover over night with starting evaporation rates in the following day of ≈1.60 kg m^−2^ h^−1^. The excellent long‐term high output performance of the solar evaporator is then attributed to the excellent self‐driving water absorption capacity based on the capillary pumping; while, the Donnan equilibrium effect ensures the low concentration of Na^+^ cations, and hence, of Cl^−^ ions within the water supplied to the evaporator's interface regulating the crystallization behavior of the salt.^[^ ^28^ ^]^ This method effectively overcomes the phenomenon of salt crystallization during the evaporation process with the consequent broader applications and prospects. Moreover, this study provides valuable insights for the practical application of polyanionic electrolyte solar evaporators for seawater desalination.

## 2 Results and Discussion

### 2.1 Fabrication and Characterization of the Solar Evaporator

**Figure 2:**
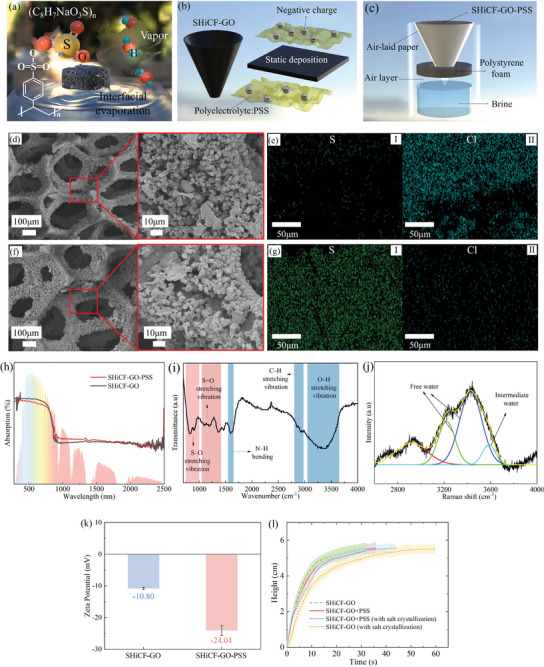
Schematics of the proposed interfacial solar evaporator principle including the fabrication and characterization of the 3D inverted conical polyanionic electrolyte assisted solar evaporator. a) Schematic diagram of the solar interface evaporation. b) PSS layer‐by‐layer static deposition functionalization method. c) Schematic diagram of the 3D inverted conical solar evaporator. d,f) SEM snapshots of the superhydrophilic copper foam SHiCF‐GO and of the superhydrophilic copper foam further funtionalised  with PSS (SHiCF‐GO‐PSS) evaporators. e,g) SEM‐EDS maps for sulfur S and chloride Cl for the SHiCF‐GO and SHiCF‐GO‐PSS solar evaporator after 9 h in brine immersion and evaporation. h) Ultraviolet‐infrared absorption spectrum of SHiCF‐GO and SHiCF‐GO‐PSS. i) FTIR diagram of SHiCF‐GO‐PSS. j) Raman spectra diagram water infiltration within the SHiCF‐GO‐PSS. k) Zeta potential results of the SHiCF‐GO and the SHiCF‐GO‐PSS. l) Capillary raise characterization of the evaporators with different configurations for a total height of 5.5 cm.

The original conical surface of the evaporator was created by bending a honeycomb pristine copper foam (PCF) into an inverted 3D conical shape with a cone's apex angle of 45°. The selected angle of 45° provides an enhanced projected area for the light incidence; while, maintaining a reasonable height for the water transport toward the top of the evaporator.^[^ ^29^ ^]^ Thereafter chemical oxidation was applied so as to create a uniform layer of copper oxide blade‐like superhydrophilic micro‐/nano‐structures decorating the micro‐structured skeleton of the PCF,^[^ ^29^, ^30^ ^]^ as represented in **Figure** **2**d. The chemical oxidation was responsible for the elaborated micro‐/nano‐blade structures, which modified the surface of the PCF, facilitating liquid spreading and transport, eventually endowing the superhydrophilic copper foam (SHiCF). Thereafter, as carboxyl groups played a positive role in assisting water supply and on light absorption, graphene oxide (GO) functionalization of the SHiCF via physical deposition yielded the SHiCF‐GO evaporator. Next, the polyanionic electrolyte layer composed of polystyrene sodium sulfonate (PSS) was adhered to the evaporator's interface through layer‐by‐layer static deposition via immersion, obtaining the unique SHiCF‐GO‐PSS reported in this work and represented in Figure 2b. The PSS formed a uniform and dense polyelectrolyte film after several deposited layers,^[^ ^31^ ^]^ which was key to achieving effective ionization desalination. Last, an insulating polyethylene foam was further placed between the evaporator and the bulk water, as represented in Figure 2c, to ensure localized heating at the evaporator's surface, minimizing heat loses toward the bulk and ensuring high evaporation efficiency.

The polyanion electrolyte film deposited on the surface of the original SHiCF‐GO solar evaporator was a long‐chain polymer with ionizable groups of PSS, minimizing the transport of Na^+^ cations; and hence of Cl^−^ ions, eventually regulating crystallization.^[^ ^32^ ^]^ Briefly, ionization salt rejection strategy based on the Donan effect referres to the implementation of a salt ion concentration barrier at the inlet of the water transport path by leveraging on the functionalized sulfonic acid groups.^[^ ^32^, ^33^ ^]^ A large number of sulfonic acid groups such as HSO3^−^ and COO^−^ aggregated at the evaporation interface after the layer‐by‐layer static deposition of the PSS, playing an important role in dissociating free Na^+^ ions and limiting the movement of Cl^−^ ions near the evaporator's inlet.^[^ ^23^, ^33^ ^]^ Figure 2d,f provides scanning electron microscopy (SEM) sharpshoots of the SHiCF‐GO and SHiCF‐GO‐PSS physical structures, for comparison. From Figure 2d,f, the addition of several layers of PSS did not contribute significantly to any micro‐ and/or nano‐structural differences between the two evaporators. The blade‐like superhydrophilic structures within the micrometer range were clearly visible in both cases, confirming that the micro‐pore structure, which plays a paramount role on liquid and vapor transport, has not been modified due to the addition of the PSS. Nonetheless, the PSS does still provide the necessary electrolyte barrier inhibiting salt ions transport and salt formation,^[^ ^3^ ^]^ as well as maintaining the necessary liquid and vapor transport for efficient solar evaporation. Moreover, the evaporator's surface light absorption is not compromised as demonstrated from ultraviolet‐infrared absorption measurements introduced below in Figure 2h. Ultraviolet‐infrared absorption spectrum shows a rather similar interface light absorption performance before (for SHiCF‐GO) and after PSS modification (for SHiCF‐GO‐PSS), with the weighted average absorption rate calculated as 93.4% and 93.2%, respectively. The absorption ratio of SHiCF‐GO is slightly higher than that of SHiCF‐GO‐PSS surface in the ultraviolet region and slightly lower in the infrared range, while their absorption and emission ratio curves almost coincide within the visible light range. Fourier transform infrared (FTIR) spectroscopy is additionally applied to identify the functional groups of the SHiCF‐GO‐PSS solar evaporator, that is, with polyelectrolyte coating, as shown in Figure 2i. When comparing the SHiCF‐GO and SHiCF‐GO‐PSS FTIR spectrums, that is, Figure S2, Supporting Information versus Figure 2i, respectively; the spectral peaks at 822 and at 906 cm^−1^ reveal the S─O stretching vibration band; while, the spectral peaks at 1100, 1180 cm, and 1370 cm^−1^ correspond to the S═O stretching vibration band. S─O and S═O stretching bands demonstrate the presence of sulfonic acid groups in the SHiCF‐GO‐PSS.^[^ ^34^ ^]^ The Raman spectrum of the O─H bond stretching frequency of the SHiCF‐GO‐PSS under water infiltration is shown in Figure 2j. The three peaks are fitted by Gaussian functions revealing the states of different types of water molecules in the SHiCF‐GO‐PSS solar evaporator. The peaks at wavelengths 3210 and 3447 cm^−1^ correspond to the ─OH expansion and tetrahedral structure of free water, while the peak at 3625 cm^−1^ is related to the weak hydrogen bonding of intermediate water exhibiting a non‐tetrahedral structure, which is conducive to the phase transition of water and the diffusion of vapor.^[^ ^20^, ^35^ ^]^

To understand the occurrence of salt deposition during continuous operation, scanning electron microscopy energy dispersive X‐ray (SEM‐EDS) spectra has been carried out on the surface of the two evaporators SHiCF‐GO and SHiCF‐GO‐PSS after 9 h of continued solar evaporation. The evaporators exhibited different analytical composition; and hence, different salt crystallization states, which are shown in Figure 2e,g. On the one hand, the SHiCF‐GO evaporator shows absence of sulfonate groups as expected and presence of Cl^−^ anions presumably attributed to salt crystals as in Figure 2e; whereas on the other hand, the significant increase in Sulphur S element after the addition of the PSS proves the successful introduction of sulfonic acid groups, while the almost absence of Cl^−^ anions indicates a significant improvement in the salt rejection ability of the evaporator as in Figure 2g. Moreover, zeta potential measurements show greater negative charges on the surface of SHiCF‐GO‐PSS, as shown in Figure 2k, which further verifies the successful addition of the PSS polyanionic electrolyte and its ionization desalination performance. Further, SHiCF‐GO‐PSS immersion in water demonstrates that during these tests, there was no significant change in the surface physical appearance nor was PSS dissolved in water, causing it to change color as per the different color of the PSS‐water solution, as reported in Figure S6, Supporting Information, which proves that PSS does not erode during long‐term immersion. We note here that other adhesion enhancing methods such as grafting may be adopted in the future, though these methods are less easy and scalable than immersion ones.^[^ ^36^ ^]^

Next, the capillary water transport ability of the evaporator after depositing PSS is further investigated by contacting and tracking the rising wetting front for the SHiCF‐GO‐PSS and SHiCF‐GO evaporators in brine at 15‰ salt concentration, that is, in between seawater and saturated solution concentrations, as reported in Figure 2l. On one hand, Figure 2l shows negligible differences in the water transport capacity between SHiCF‐GO‐PSS and SHiCF‐GO, which indicates that the PSS does not affect the physicochemical properties of the evaporator. Such water transport characterization confirms the hydrophilicity/superhydrophilicity of the SHiCF‐GO‐PSS surface. On other hand, after 10 days of evaporation experiments, the capillary raise within the SHiCF‐GO‐PSS interface still maintains a high‐water transport without interference of salt crystallization with results agreeing within the error bounds of the original evaporator whereas the water transport capacity of the SHiCF‐GO is reduced as shown in Figure 2l.

### 2.2 Terminal Salt Precipitation Induced by Water Transport Mode

**Figure 3:**
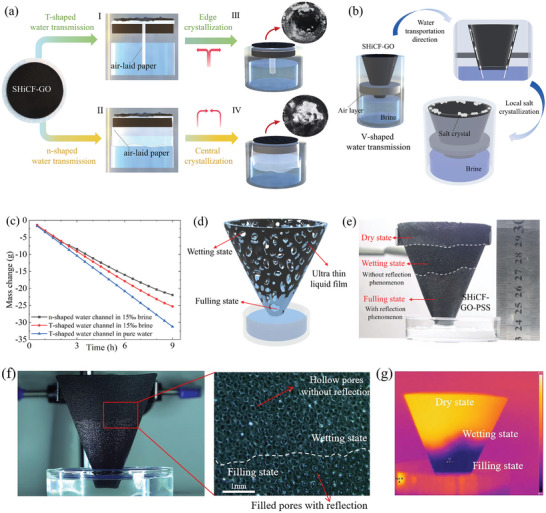
Local salt crystallization caused by the different water transport mechanisms. a) Water transport mechanism (I) and salt‐out image (III) for a T‐shaped water path evaporator, and water transport mechanism (II) and salt‐out image (IV) for an n‐shaped water path evaporator. b) Schematic diagram of the V‐shaped 3D conical water transport path evaporator. c) Mass change (g) for (squares) n‐shaped and (circles) T‐shaped evaporators in brine and (triangles) T‐shaped evaporator in pure water. d) Schematics of water wetting and wicking within the superhydrophilic pores and along the conical evaporator. e) Actual snapshot highlighting the water wetting and wicking film exemplified by the fully wetted, moist, and dry areas of the evaporator. f) SHiCF‐GO‐PSS porous skeleton snapshot highlighting the wetted pores reflecting light and the hollow pores still not filled with seawater. g) Infrared imaging snapshot showing the wetting front within or along the SHiCF‐GO‐PSS porous skeleton exhibiting three different clear states: filling, wetting, and dry states present during the capillary rising process reported in Figure 3e.

The water transport path plays a very important role in solar evaporators; however, prolonged high‐intensity evaporation with water transport ensuing in a fixed direction can lead to salt crystallization and accumulation hindering sunlight absorption, and liquid and vapor transport eventually penalizing evaporation rates and efficiency.^[^ ^25a^ ^]^ In some extreme cases, the structure of the solar evaporator may be even damaged during salt crystallization and accumulation.^[^ ^37^ ^]^ To better understand how water is transported from the bulk brine to the evaporation interface, and to justify our decision of making use of the V‐shaped configuration, three different evaporator water paths are considered: a T‐shaped, an n‐shaped, and a V‐shaped configuration. On the one hand, the T‐shaped evaporator is designed to transport water upward to the center of the evaporator and then sidewise toward the outer edges of the evaporator, preventing salt accumulation in the center of the evaporator as shown in **Figure** **3**a‐I and 3a‐III. On the other hand, the n‐type evaporator transports the water upward towards the outer edges of the evaporator and then toward the center of the evaporator where most of the salt crystallization occurs, as shown in Figure 3a‐II and 3a‐IV. Hence, the direction of the water delivery path and the deposition of the salt crystals can be adjusted via the evaporator's shape and structure so as to achieve optimal balance between evaporation and crystallization. More details on the direction of the water transport and preferential regions for salt crystallization function of the shape of the evaporator transmission for the T‐shaped and n‐shaped sections can be found in Section S8, Supporting Information: Salt crystallization behavior of evaporators with different configurations. As shown in both Figure S7, Supporting Information; Figure 3a‐III and 3a‐IV, a significant difference in the coverage area distribution of the salt crystals between the T‐type evaporator and the n‐type evaporator is evident.

As conveyed above, depending on the configuration of the evaporator, salt accumulation occurs preferentially on different regions without covering the entire evaporation surface. This points out that salt discharge from the solar evaporator may be achieved by changing the direction of the water delivery.^[^ ^38^ ^]^ In the T‐shaped evaporator, capillary force drives water flow to transfer ions from the bulk liquid to the appropriate edge positions where salt crystallization does not affect greatly the performance of the evaporation interface.^[^ ^9b^ ^]^ Crystallization occurs preferentially at the edges because in the T‐shape, configuration evaporation is higher at the periphery than that at the center, and as such the concentration of brine at the edge reaches saturation first prioritizing salt precipitation.^[^ ^39^ ^]^ Such salt precipitation additionally accelerates water transport speed from the center to the periphery supporting the greater salt accumulation at the surface edges as shown in Figure 3a‐III. In contrast, in the n‐type shaped evaporator, the water transport path occurs through the skirt shaped bottom of the evaporation interface toward the outer edges or periphery of the evaporator and then toward the center. In this configuration, the density of the brine is higher at the center than at the periphery reaching saturation and prioritizing salt precipitation resulting in salt accumulation at the center of the evaporation instead.^[^ ^40^ ^]^ Here, salt crystals gradually build up from the inside toward the outside. While it is possible to control the preferential deposition of salt crystals based on how the brine is transported into the evaporator surface, the salt self‐removal performance of either the T‐shape or the n‐shape evaporators is negligible because gravity cannot ease the removal of crystals on such horizontal configurations. In both configurations, salt crystals eventually cover the entirety of the evaporation interface blocking the pores, which cause great adverse effects on both solar light absorption, vapor diffusion, and water transport. Therefore, the evaporation rate gradually weakens with salt crystallization, which is further diminished in the case of the n‐shaped evaporator when compared to the T‐shaped one, which is demonstrated by the non‐linear change of the brine mass in time opposed to the linear change of pure water mass in time as represented in Figure 3c. To overcome the limitations on salt self‐removal in the case of the horizontal T‐type and n‐type evaporators, the V‐type or 3D cone evaporator represented in Figure 3b is adopted. In this configuration, crystals form at the edge and then fall once gravitational forces pulling the crystals downwards overcome their adhesion providing a path for self‐removal of crystals as earlier reported.^[^ ^7^, ^10^ ^]^ To further confirm the occurrence of a thin water film along the evaporator interface, Figure 3d,e includes the schematics and a snapshot of the actual water wetting and wicking within the superhydrophilic pores and along the conical evaporator exemplified by the fully wetted, moist, and dry areas of the evaporator. We also note here that in the absence of a wetting film and evaporative cooling, the evaporator interface temperature would increase to values further than the ones reported during contentious operation near 32 °C, as reported in Figure 7b. Next, Figure 3f,g provide further observations of the interactions between the wicking water and the porous skeleton of the SHiCF‐GO‐PSS evaporator. Figure 3f in particular clearly shows, via the reflected light, the occurrence of a wetting film with presence of water trapped within the pores of the evaporator below the wetting film and hollow pores with absence of water above the wetting film. Moreover, infrared thermography can also be utilized to retrieve stratification of the seawater state around the skeleton during the spreading process of the wetting front from the perspective of temperature changes. Figure 3g shows then via color stratification, the boundary among the filling state, the wetting state, and the dry state. It is worth noting that similar observations to those reported in Figure 3g have been utilized in the past to verify the occurrence of the wetting film on their bridge‐arch solar evaporator in the work of Zou et al., amongst others.^[^ ^41^ ^]^ More detailed information on the characterization of the wetting front can be found in Section S13, Supporting Information: Wettability and Capillary Raising Characterization and within SI Video 1 within the accompanying Supplementary Information.

To further confirm the relationship between salt accumulation and water transport path across the superhydrophilic porous structure, the inverted SHiCF‐GO cone evaporator is placed in brine for 10 days. The V‐shaped 3D conical evaporator exhibits similar salt accumulation resistance as the T‐shaped evaporator where salt crystals are only observed at the top edge of the evaporator; while, almost no visible salt crystals are observed on the inner and outer walls of the cone as represented in schematics in Figure 3b.^[^ ^42^ ^]^ This is due to its strong water absorption and upward transport ability owed to the preferential evaporation and salt crystallization at the cone top edges in a similar manner as it occurred for the T‐shaped evaporator with the additional occurrence of crystals self‐cleaning via gravitational forces in the V‐shaped evaporator.^[^ ^38^, ^43^ ^]^ Importantly, under no sunlight irradiation, almost all salt crystals return to the bulk brine over the 10 h soak period as shown in Figure S9, Supporting Information. This is due to the continuous water absorption endowing the evaporator with excellent self‐clean behavior and impressive cycling stability. Controlling the position of the salt crystallization is hence a positive strategy in the design of evaporators to continuously generate vapor and simultaneously harvest salt.^[^ ^2^, ^8^ ^]^ By carefully controlling the flow direction and evaporation area of the water supply, salt crystallization can be limited to specific local areas.^[^ ^4^, ^19^ ^]^ This method of isolating salt crystals outside the evaporation area can maintain a high evaporation performance; while at the same time, achieving efficient salt collection.

### 2.3 Donnan Equilibrium and Ionization Salt Removal Strategy

**Figure 4:**
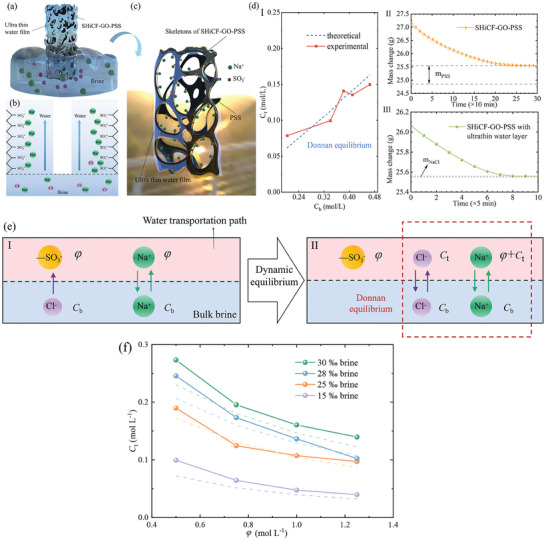
Schematic diagram and working principle of the polyelectrolyte preventing salt crystallization. a) Schematic and b) detailed diagram of polystyrene sodium sulfonate PSS fundamentally blocking Cl*^−^* anions transport; and hence, salt crystallization. c) Schematic diagram of Donnan equilibrium. d) Experimental and theoretical results on I) Donnan equilibrium based on Equation (1), II) PSS mass measurement, and III) limited Na^+^ and Cl^−^ mass measurement. e) Schematics of the dynamic process of Donnan equilibrium induced by the polyelectrolyte where (bottom) shaded blue and (top) shaded red represents the bulk brine and the vicinity of the evaporator's interface I) out of equilibrium and II) in equilibrium. f) The relationship between the concentration of Na^+^ or Cl^−^ ions that diffuses into the water transport path *C*_t_ function of the concentration of sulfonic acid ions and salt ions in the water supply path *φ* for different brine concentrations *C*_b_, the solid lines with dots is data points are the experimental results, and the dotted lines are the theoretical estimation results based on Equation (1).

The cone shape of the evaporator eases salting‐out via localized salt crystallization at the edges; however, it does not guarantee the continuous steady state performance of the evaporator during long‐term operation. To overcome such deficiency, the salt free evaporation system SHiCF‐GO‐PSS is proposed, which consists of a top solar absorber, collaborative water liquid and vapor mesoporous conveying paths, a bottom insulation polymeric bracket, and polyanionic electrolyte functionalization as earlier introduced and shown in Figure 1. The polyanionic electrolyte layer exploits the Donnan effect where fixed charged anions sulfonic acid groups such as ─SO_3_H and COO^−^ aggregate can confine counter ions within the water supply path such as potasium K^+^ or sodium Na^+^ as per the expected electrically neutral nature of the solution near the polyanionic electrolyte, as represented in **Figure** **4a**.^[^ ^44^ ^]^ First, upon immersion in brine, the sodium cations present in the polyanionic electrolyte polystyrene sodium sulfonate ─SO_3_Na dissociate from the polystyrene sodium sulfonate SO_3_^−^. The presence of these charged polystyrene sulfonate anions generates high chemical potentials at the outermost layer of the evaporator eventually altering the distribution balance of salt ions between the bulk saltwater and the water supply pathways. Then, in addition to the Na^+^ cations already present in the bulk, further amount of Na^+^ cations distribute along the polyanionic electrolyte polystyrene sulfonate around the skeleton of the evaporator, as in Figure 4b,c, eventually inhibiting the transport of Cl^−^ anions; and hence, of salt crystallization. The ─SO_3_Na termination at the evaporator's interface is further confirmed by energy dispersive X‐ray spectroscopy (EDS) in Figure 2g and Fourier transform infrared (FTIR) spectroscopy in Figure 2i.^[^ ^22a^ ^]^ From FTIR spectroscopy in Figure 2i, the peaks located at 822, 906, 1100, and 1180 cm^−1^ correspond to the S─O and S═O stretching vibration bands in sulfonic acid and sulfonic acid salts; while, EDS spectrum in Figure S3, Supporting Information confirms the presence of uniformly distributed Na^+^ cations in the absence of Cl^−^ element, as earlier shown in Figure 2g.^[^ ^45^ ^]^

Hence, the high chemical potential generated by the restricted Na^+^ cations induces the Donnan equilibrium distribution at the interface between the water supply path and the bulk brine,^[^ ^46^ ^]^ which reduces the diffusion of Cl^−^ anions, minimizing local salt concentration. The dynamic process of the Donnan equilibrium induced by a sulfonate or a polyelectrolyte is sketched in Figure 4e. At the early stages of contact between the water supply path of SHiCF‐GO‐PSS and the bulk brine, the ion concentration inside and outside the contact surface varies with small ions moving freely.^[^ ^47^ ^]^ The large PSS molecules are immobilized on the surface of the micro nano structured skeleton; so to avoid their diffusion into the bulk brine, the Na^+^ ions dissociate in the bulk. The hydrolyzed or dissociated Na^+^ ions then become easily attracted by the ─SO_3_^−^ due to the chemical bond attraction, making them only able to move weakly around the sulfonic acid group.^[^ ^46^ ^]^ During the process of water being pumped from the brine to the supply path and through the skeleton, Na^+^ and Cl^−^ ions in the brine can freely move and accumulate within the liquid film, resulting in unequal ion concentrations between the liquid film and the bulk brine. However, the Na^+^ interacting with the ─SO_3_^−^ cannot diffuse into the bulk brine, but the ions in the bulk brine solution can accumulate near the polystyrene sulfonate interface, resulting in a charge equilibrium where the ion concentrations inside and outside the water supply interface are not equal, but the product of anion and cation concentrations is equal near the PSS, as sketched in Figure 4a,e. The minimization of Cl^−^ anions diffusion enables brine evaporation and absence of salt crystallization for long periods of time without compromising the energy efficiency or evaporation rates. The Donnan equilibrium formula establishes the then quadratic relation between the ion concentration in the water transport path and the ion concentration in the bulk as follows:^[^ ^23^ ^]^

(1) $$C_{t}\left(\varphi +C_{t}\right)={C_{b}}^{2}$$

where *φ* is the concentration of the confined anion (such as R–SO_3_^−^) and counter‐ion (such as Na^+^) in the water transport path, *C*_t_ refers to the Na^+^ or Cl^−^ ion concentration that diffuses into the water transport path, and *C*_b_ is the salt concentration in brine. Consequently, the amount of salt ions diffusing into the water transport/supply path (*C*_t_) can be regulated by the concentration of the confined counter‐ions *φ*. For this specific system, from Equation (1) as well as experimental measurements, the ion concentration within the diffusion path can be as low as 0.08 and 0.15 mol L^−1^ for brine concentrations as high as 0.15 and 0.48 mol L^−1^, respectively, as represented in Figure 4d, which demonstrates the lower concentration of Cl^−^ anions within the water transport path. Complementarily, for a given concentration of the brine *C*_b_, the Donnan effect establishes a decrease in the concentration that diffuses through the polyanionic electrolyte layer *C*_t_ as the concentration of confined anions *φ* increases, which is represented in Figure 4f. In essence, the Donnan equilibrium effect in solar evaporators constrains the Na^+^ cations in the presence of the polystyrene sulfonate, leading to only trace amounts of Cl^−^ anions being able to move to the water supply path of the evaporator, minimizing the likelihood of salt crystallization.^[^ ^48^ ^]^

To further verify the equilibrium of the Donnan effect, the Na^+^ and Cl^−^ distributions between SHiCF‐GO‐PSS and bulk brine are measured through mass change experiments, for which specific details and steps can be found in the accompanying Sections S11 and S12, Supporting Information. As shown in Figure 4d, the concentrations of restricted Na^+^ (*C*_t_) calculated based on the Donnan equilibrium formula are 0.08 mol L^−1^ (in 15‰ brine), 0.10 mol L^−1^ (25‰ brine), 0.14 mol L^−1^ (28‰ brine), 0.14 mol L^−1^ (30‰ brine), and 0.15 mol L^−1^ (36‰ brine), respectively. Comparing the experimental and theoretical value of *C*_t_, it is confirmed that there exists a Donnan distribution equilibrium at the interface between the SHiCF‐GO‐PSS solar evaporator and the bulk brine (Figure 4b). Therefore, PSS effectively reduces the salt ion concentration (*C*_t_) in the water transportation path, as shown in Figure 4f, demonstrating the salt formation resistance of our evaporator based on the Donnan effect.

In order to evaluate the salt resistance conferred by the Donnan effect, the SHiCF‐GO without PSS functionalization is used as control. In a conventional solar evaporator, it is evident that as water evaporates, salt ions accumulate and eventually crystallize at the evaporator's interface.^[^ ^49^ ^]^ SEM‐EDS element map after 9 h in brine immersion and evaporation in Figure 2e confirms the presence of Cl^−^ and absence of S, which is attributed to the presence of salt crystals. The accumulation of salt not only reduces sunlight absorption but also blocks the path of water supply and vapor diffusion, thereby reducing evaporation performance.^[^ ^2^, ^32^, ^50^ ^]^ In contrast, no salt accumulation after 9 h in brine immersion and evaporation is observed on the salt resistant solar evaporator functionalized with PSS, that is, SHiCF‐GO‐PSS, which is confirmed by the absence of Cl^−^ and presence of S by the SEM‐EDS element map presented in Figure 2g.

Next, we evaluate experimentally the evaporation performance under continuous operation in 15‰ brine under 1‐Sun irradiation. The rather constant evaporation rates reported over extended periods of time of up to 10 days indicate that the solar evaporator designed based on the Donnan effect SHiCF‐GO‐PSS has good salt resistance performance, which is thoroughly introduced and discussed in the next subsection.

### 2.4 Evaporation Performance of the Polyelectrolyte Evaporator

The continuous operation without penalizing the evaporation rate performance is a necessary condition for the implementation of solar evaporators from brine in industrial applications. The evaporation rate and dark evaporation rate for all the different configurations of evaporator systems investigated in this work on the 1st day and after 10 days of continuous operation, along with the % decrease in the evaporation rates, can be found in **Table** **1** below.

**Table 1:** Evaporation rates for the different configurations. Evaporation rate (kg m^−2^ h^−1^) on Days 1 and 10, the dark evaporation rate (kg m^−2^ h^−1^), and % decrease in evaporation rate between Days 1 and 10 for the different configurations of evaporation systems.

Evaporation system	Evaporation rate on DDay 1 [kg m^−2^ h^−1^]	Evaporation rate on day 10 [kg m^−2^ h^−1^]	% Decrease evaporation rate between Days 1 and 10 [%]	Dark evaporation rate [kg m^−2^ h^−1^]
Brine	0.13	—	—	0.07
SHiCF	1.14	0.87	23.7	0.11
SHiCF‐GO	1.39	0.94	32.4	0.12
SHiCF‐GO‐PSS	1.44	1.42	1.4	0.09
SHiCF‐GO + air‐laid paper	1.67	1.35	19.2	0.08
SHiCF‐GO‐PSS + air‐laid paper	1.68	1.62	3.6	0.09
SHiCF‐GO‐PSS + air‐laid paper‐PSS	1.68	1.67	0.6	0.08

From Table 1, the rather large differences in the evaporation rates in the absence of the PSS functionalization when comparing Day 1 to Day 10 with up to 32.4% detriments on the evaporation rates are highlighted. Nonetheless, in the presence of the PSS functionalization, a maximum decrease of 3.6% is reported in the case of the SHiCF‐GO‐PSS+air‐laid paper evaporator; while if the air‐laid paper is further functionalized, a less than 1% decrease in the evaporation rate is reported.

**Figure 5:**
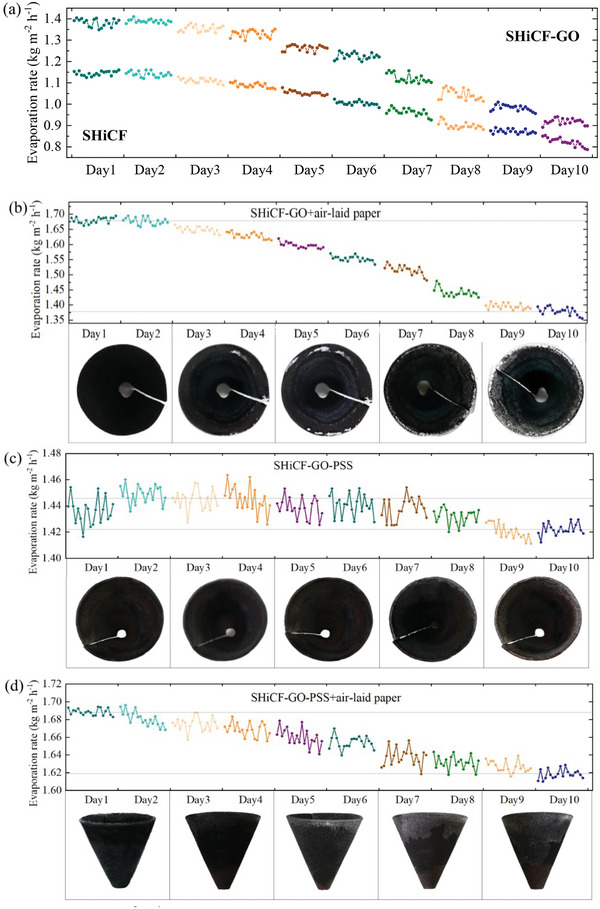
Evaporation rate (kg m^−2^ h^−1^) function of time (days) and evaporator surface snapshots showing salt crystallization phenomenon for different evaporator configurations within one cycle, that is, Day 1 to Day 10 for a) SHiCF and SHiCF‐GO, b) SHiCF‐GO+air laid paper, c) SHiCF‐GO‐PSS, and d) SHiCF‐GO‐PSS+air laid paper.

Next, the evolution of the evaporation rates in time from Day 1 to Day 10 for SHiCF, SHiCF‐GO, SHiCF‐GO+air‐laid paper, SHiCF‐GO‐PSS, and SHiCF‐GO‐PSS+air‐laid paper, including snapshots of the evaporator surface at different instants of time, are included in **Figure** **5**.

When drawing our attention to the different configurations performance reported in Table 1 and Figure 5, in the absence of air‐laid paper and/or PSS functionalization, the evaporation rates of the SHiCF and the SHiCF‐GO without air‐laid paper, that is, Figure 5a and Table 1, significantly decrease after a 10‐days operation in 15‰ NaCl solution. A decrease from 1.14 to 0.87 kg m^−2^ h^−1^ and from 1.39 to 0.94 kg m^−2^ h^−1^ is reported on SHiCF and SHiCF‐GO, respectively, which are a 23.7% and a 32.4% decrease in the evaporation rates. At the beginning of a 10‐days cycle, that is, after 9 h of salt evaporation, the average evaporation rate of SHiCF‐GO is much higher than that of SHiCF, which is owed to greater broadband solar light absorption ability and additional sorption capabilities of the GO.^[^ ^28^, ^51^ ^]^ The combination of the inherent wide range and strong light absorption of the GO sheets with the cumulative effect generated by the honeycomb multi‐layer stacking morphology ensure effective and extensive light absorption throughout the entire wavelength range of the sunlight. However, it is worth noting that as evaporation continues, the difference in evaporation rate begins to significantly decrease from Day 4 onward. While the evaporation rates of both SHiCF‐GO and SHiCF systems gradually decrease, the difference in daily evaporation rates between the two systems gradually narrows from 0.25 to 0.07 kg m^−2^ h^−1^. This can be attributed to the accumulation of salt crystals on the evaporation surface, which blocks the GO or the micro‐/nano‐pores. Such salt accumulation hinders any earlier evaporation rate benefit empowered by the GO with the consequent decrease in device performance and shortened lifespan.^[^ ^52^ ^]^

In the case of the solar evaporator SHiCF‐GO wrapped with air‐laid paper and reported in Figure 5b, the average net solar evaporation rate initially reaches ≈1.67 kg m^−2^ h^−1^ and begins to gradually decay down to 1.35 kg m^−2^ h^−1^ after 10 days, which is a 19.2% decrease in the evaporation rate after Day 3. This is ultimately due to severe salt accumulation as shown in snapshots of the evaporator taken from the top in Figure 5b. It is then clear that in the absence of PSS, salt crystals as a salt ring that extends from the outer to the inner region of the evaporator ensues as salt accumulates, which are visible after Day 3. In this SHiCF‐GO+air laid paper evaporator, the brine is transported by capillary action from the bulk of the solution to the bottom of the evaporator through the air‐laid paper, and then toward the top of the evaporator relying on its superhydrophilicity.^[^ ^53^ ^]^ As water evaporation is favored by higher temperatures and thin salt films, crystallization is hence preferential at the top of the evaporator forming a salt ring along the edge. The large amount of salt crystals on the top of the circular ring and the top of the air‐laid paper block the water supply path as well as the light absorption. The salt crystallization pattern of the SHiCF and the SHiCF‐GO is similar to that of the SHiCF‐GO wrapped with air‐laid paper, where the top salt concentration ultimately exceeds its solubility limit in water, leading to salt crystallization and the formation of the observed salt rings. As the salt ring continues to spread downwards, salt crystals eventually block the pores for both liquid and vapor transport taking place during evaporation with the consequent detriment in performance from 19.2% in 10 days.

Therefore, to fundamentally prevent the accumulation of salt and the consequent reduction in evaporation rates, regulating the salt concentration in the water transport path is key. In the presence of PSS functionalization, that is, SHiCF‐GO‐PSS without air‐laid paper, solar evaporator in brine environment shows no obvious presence of salt crystals on the surface even after 6 days of continuous operation as depicted in Figure 5c. In this configuration, the net evaporation rate of the evaporator remains relatively stable for 7 consecutive days of desalination, with values ranging from 1.40 to 1.44 kg m^−2^ h^−1^. However, the evaporation efficiency shows a significant decrease starting from Day 8; although, the evaporation rate remains at ≈1.42 kg m^−2^ h^−1^ on Days 9 and 10, which is a less than 1.4% decrease when compared to the initial evaporation rate. This excellent preservation of the evaporation performance is undoubtedly attributed to the exploitation of the Donnan equilibrium between the water supply path and the bulk brine, which limits the upward movement of Cl^−^ ions fundamentally inhibiting the formation of salts. At the beginning of the cycle, a small amount of Cl^−^ ions enters the water supply path without nucleating or crystallizing.

Despites the good performance over time reported for the SHiCF‐GO‐PSS evaporator, the evaporation rate could be further enhanced by the introduction of the air‐laid paper with and without PSS functionalization. The evaporation structure with the addition of an air‐laid paper exhibits a high‐performance supplementary water transport effect, which is attributed to the synergistic effect of the capillary pump of the air‐laid paper polyester fibers and that of the superhydrophilic skeleton. A 10‐day evaporation test is conducted to verify the stability performance of the SHiCF‐GO‐PSS+air‐laid paper evaporator, which is shown in Figure 5d. With the assistance of the air‐laid paper, the evaporation rate increases by 16.7% when compared to SHiCF‐GO‐PSS without air‐laid paper, maintaining a 1.68 kg m^−2^ h^−1^ evaporation rate on Day 1 and Day 2. However, the evaporation rate gradually decreases from Day 2 onward, which proves that the addition of air‐laid paper without functionalization eventually reduces the continuous high output of the evaporator. Although the air‐laid paper improves the rate of water supplement to the copper foam cone, it is worth noting that the air‐laid paper without functionalization enables salt crystallization decreasing the pores size as well as the water layer at the evaporation interface. Nonetheless, the reduction in the evaporation reported in this case is less than 4%. From the sideview snapshots of the evaporator provided in Figure 5d, the continuous precipitation of salt crystals from top to bottom covering almost half of the area on the air‐laid paper prevents evaporation effectiveness. Crystals adhere at the interface between the air‐laid paper and the outer wall of the evaporator, blocking the vapor diffusion path and the transport of water molecules from the air‐laid paper to the SHiCF‐GO‐PSS skeleton, causing irreversible drop of the evaporation rate performance. Different from the top salt ring observed in the case of SHiCF‐GO in the presence of an air‐laid paper without functionalization shown in Figure 5b, in this case salt crystals form on the outer wall of the evaporator without visible crystalline salt layer formed on the inner wall, which confirms that the salt layer on the outer wall is not due to passive desalting of the SHiCF‐GO‐PSS evaporator outermost surface. On the one hand, the crystallization of salts on the outer wall is attributed to the overflow salt ions within the air‐laid paper; whereas, on the other hand, there is a large number of sulfonic groups at the contact surface between the air‐laid paper and the copper foam, which inhibits the transportation of Cl^−^ ions from the air‐laid paper to the ultra‐thin water layer on the superhydrophilic framework, resulting in a large number of crystalline salts staying at the interface, and then adhering to the air‐laid paper outer wall.

**Figure 6:**
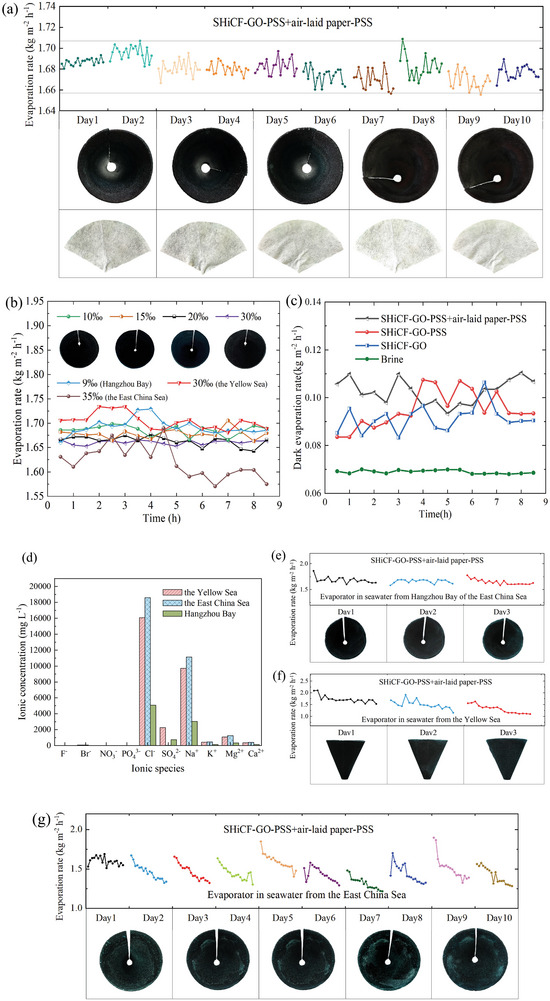
Evaporation rate (kg m^−2^ h^−1^) function of time (days) and evaporator surface snapshots of the PSS functionalized polyelectrolyte evaporator under different conditions. a) Evaporation rate and salt crystallization phenomenon in time of SHiCF‐GO‐PSS+air‐laid paper‐PSS evaporator system for one 10‐days cycle including evaporator and white air‐laid paper photographs where the spots in the air‐laid paper are shadows. b) Evaporation rate in time and evaporator snapshots of the SHiCF‐GO‐PSS+air‐laid paper‐PSS in brine at different salinity and real seawater. c) Dark evaporation rate for the different configurations of evaporators. d) Types and concentrations of ions in seawater from Hangzhou Bay in the East China Sea and from the Yellow Sea. e) Evaporation rate and salt crystallization phenomenon in time of SHiCF‐GO‐PSS+air‐laid paper‐PSS evaporator system in seawater from Hangzhou Bay in the East China Sea. f) Evaporation rate and salt crystallization phenomenon in time of SHiCF‐GO‐PSS+air‐laid paper‐PSS evaporator system in seawater from the Yellow Sea. g) Evaporation rate of SHiCF‐GO‐PSS+air‐laid paper‐PSS evaporator systems in seawater from the East China Sea.

In order to minimize the salt crystal formation on the outermost surface of the evaporator, the air‐laid paper wrapping the SHiCF‐GO‐PSS was further functionalized with PPS, that is SHiCF‐GO‐PSS+air‐laid paper‐PSS, providing a stable evaporation rate between ≈1.66 and 1.71 kg m^−2^ h^−1^ throughout 10 days of continuous operation, which is a less than 1% decrease in performance, as shown in **Figure** **6a** and Table 1. As expected, the deposition of the PSS polyelectrolytes onto the air‐laid paper also serves as a barrier blocking Cl*^−^* ions from wicking or diffussing into the evaporator as well as into the air‐laid paper, which is confirmed by the absence of salt crystals in both the evaporator as well as the air‐laid paper. Specifically, the white air‐laid paper and the evaporator cone do not discolor or show the presence of salt crystals even after 10 days of solar evaporation as shown in Figure 6a, indicating the excellent salt ions blocking effect of the PSS polyelectrolyte evaporator eventually hindering the formation of salt crystals. The formation of crystals is clearly inhibited by the presence of sulfonic acid groups blocking a large amount of dissolved Na^+^ and Cl^−^ ions within the bulk brine from entering the evaporator supply path empowered by the Donnan effect, which minimizes the salt crystals aggregation at the interface between the air‐laid paper and the evaporator ensuring the smooth transfer of water molecules. Nonetheless, within real seawater, there are further ions in solution other than Na^+^ and Cl^−^, which may play a detrimental role on the evaporator performance and evaporation rates robustness.^[^ ^54^ ^]^ To demonstrate the potential of the polyanionic electrolyte evaporator proposed here for practical applications, evaporation tests under real seawater from Hangzhou Bay in the East China Sea (121°55´, 30°81´), the Yellow Sea (120°37´, 36°05´), and the East China Sea (125°42´, 30°17´) were used. All the seawater from the East China Sea and from the Yellow Sea contain other ions in solution such as: SO_4_^2−^, K^+^, Mg^2+^, Ca^2+^, F^−^, Br^−^, NO_3_^−^, and PO_4_^3−^, as detected via ion chromatography and represented in Figure 6d with photos of seawater undergoing sedimentation are shown in Figure S12, Supporting Information. From Figure 6d, the concentration of Mg^2+^ ions is only 11% that of the Na^+^ ions concentration, and even lower concentrations of K^+^ and Ca^+2^ are found. The concentration of other anions such as F^−^, Br^−^, NO_3_^−^, and PO_4_^3−^ ions is extremely low. When comparing the different real seawater sources, the seawater from the Yellow Sea contains twice or even three times greater ion concentration of the elements analyzed and reported in Figure 6f. Evaporation tests in time were carried under seawater from the Hangzhou Bay in the East China Sea with a salinity of 9‰ for three days; the evaporator maintained a stable evaporation rate of 1.63–1.68 kg m^−2^ h^−1^ in the almost complete absence of salt accumulation on the surface of the SHiCF‐GO‐PSS evaporator with air‐laid paper‐PSS, as shown in Figure 6e. Only the faint presence of salt crystals could be detected only at the top rim of the evaporator, verifying the feasibility of the proposed evaporator to suppress salt accumulation/crystallization under real practical operation. Similarly, evaporation tests carried out under seawater from the Yellow Sea with a salinity of 30‰ and up to 2 to 3‐fold greater amount of ions in solution for 3 days showed a slight decrease in the Yellow Sea seawater evaporation rate and slight occurrence of salt crystals as shown in Figure 6f. A ten day evaporation experiment in seawater from the East China Sea with salinity of 35‰ showed that the evaporation rate of the East China Sea seawater was in the range of 1.23–1.61 kg m^−2^ h^−1^, and visible salt crystals appeared on the fourth day, as shown in Figure 6g. For high salinity seawater in the East China Sea, as the water evaporated rapidly, more metal ions or unknown impurities diffused into the water supply channels, resulting in precipitation or retention on the surface of the absorbent body in the pore channels. At this point, the surface of the evaporator with salt deposition could not fully absorb and utilize sunlight, resulting in a slight decrease in the efficiency of photothermal steam conversion. The slightly worse performance reported under the Yellow Sea and the East China Sea seawater conditions is attributed to the greater stresses imposed by the larger accumulation or crystallization of ions within the lower part of the evaporator. To counteract this decay in performance under high ions concentrations, future work may focus on the use of different polyelectrolytes with higher ionic strength or increasing the polyelectrolyte density. These actions should appropriately regulate the ion concentration at the bulk seawater near the evaporator intake, minimizing the transport of ions to the evaporator and hence any ion accumulation or salt crystallization.

To further investigate the application performance of the combination of air‐laid paper‐PSS and SHiCF‐GO‐PSS and the evaporators’ feasibility in higher concentration brine environments, the evaporation rate on brines and real seawater varying in concentrations from 10‰ to 30‰ during 9 h operation are reported in Figure 6b. After 9 h of continuous evaporation, no appreciable decay on the evaporation rate was observed independently of the brine concentration utilized, which ranged between 1.65 and 1.70 kg m^−2^ h^−1^. In addition, snapshots of the evaporator after 9 h also shown in Figure 6b as insets did not show any significant salt precipitation at the evaporator's inner surface or edges. Under a dark environment, the implementation of an evaporator increased the evaporation rates when compared to no evaporator as shown in Figure 6c. In addition, unlike under sun illumination, the evaporation rates when comparing SHiCF‐GO, SHiCF‐GO‐PSS, and SHiCF‐GO‐PSS+air‐laid paper‐PSS remained rather constant over 9 h of operation with values self‐contained between 0.08 and 0.11 kg m^−2^ h^−1^.

### 2.5 Thermal Management Mechanisms and Energy Utilization Efficiency

**Figure 7:**
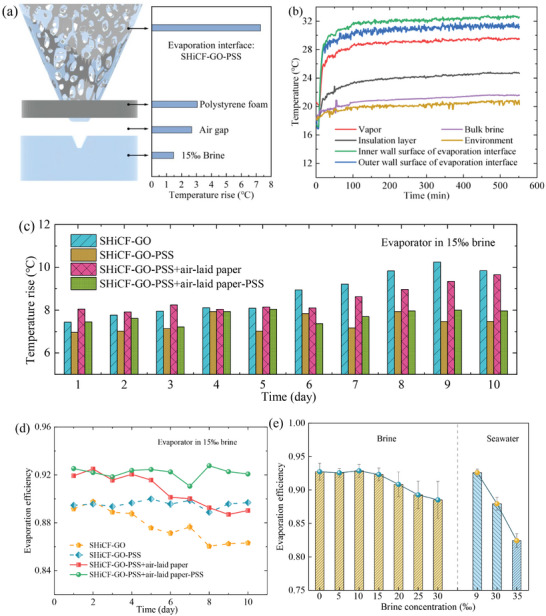
Thermal management of the different evaporators in 15‰ brine concentration. a) Temperature increases (°C) of each layer between the start and the end of the experiment for the SHiCF‐GO‐PSS evaporator, that is, during 9 h of operation. b) Temperature distribution (°C) in time (s) at various zones of the SHiCF‐GO‐PSS evaporator. c) Evaporation interface temperature rise (°C) function of time (days) for SHiCF‐GO, SHiCF‐GO‐PSS, SHiCF‐GO‐PSS+air‐laid paper, and SHiCF‐GO‐PSS+air‐laid paper‐PSS evaporators for a 10‐days cycle. d) Average evaporation efficiency (%) function of time (days) for SHiCF‐GO, SHiCF‐GO‐PSS, SHiCF‐GO‐PSS + air‐laid paper, and SHiCF‐GO‐PSS + air‐laid paper‐PSS evaporators for a 10‐days cycle. e) Evaporation efficiency of SHiCF‐GO‐PSS + air‐laid paper‐PSS in different salinity water environments from 0‰ to 30‰ for a 10‐days cycle and real seawater with the salinity of 9‰ (Hangzhou Bay in the East China Sea), 30‰ (the Yellow Sea) for a 3‐days cycle, and 35‰ (the East China Sea) for a 3‐days cycle.

The excellent evaporation rates reported rely on the high thermal conductivity of the copper foam framework maintaining uniform temperatures along the evaporator's interface coupled to the insulating polyethylene foam allowing for heat localization at the evaporator's interface and not to the bulk brine of 15‰. Hence, most of the absorbed solar energy is then localized at the solar evaporator's interface for effective water evaporation with the associated mitigation of heat loses otherwise transferred to the bulk saltwater. As water evaporates, the latent heat of vaporization is removed from the system regulating the system's temperature depending on its configuration. Next, **Figure** **7** analyzes the thermal performance and heat transfer mechanisms of the SHiCF‐GO‐PSS under 1‐Sun irradiation, which involves several processes, including heat losses to the environment from radiation (4.7%) and from convection (3.0%), as well as to the underlying 15‰ brine via conduction (1.2%), as shown in Table S2, Supporting Information. The evaporation efficiency ranges then from 92% at 0‰ brine concentration down to 89% for 30‰. The low heat losses are attributed to the significant reduction in conduction and convective heat losses between the evaporator and the bulk brine or the environment. To this end, the polyethylene foam thermally isolates the evaporator significantly reducing the total heat loss of the evaporator toward the bulk, which is owed to the air insulation layer with a thermal conductivity one order of magnitude smaller than that of water, that is, 0.025 W m^−1^ K^−1^ versus 0.62 W m^−1^ K^−1^. The photothermal surface ensures the uniform temperature distribution along the evaporators surface.

In addition, effective vapor diffusion upon evaporation has a positive impact on improving energy utilization efficiency. To better understand the temperature distribution within the evaporator, the temperature at each structural layer (the inner and the outer wall surface of the evaporator, the insulation layer, the vapor, the environment, and the bulk brine) during operation is measured in real‐time by thermocouples over a period of 9 h for the SHiCF‐GO‐PSS, as shown in Figure 7b; while, temperature increases between the start and after 9 h of operation are reported in Figure 7a. Under 1‐Sun irradiation, the temperature of the bulk brine only increases by 1.5 °C within 9 h for the SHiCF‐GO‐PSS system. In the typical evaporation system with a flat disk evaporator, the temperature of the evaporation surface is 37.3 °C^[^ ^29^ ^]^ compared to 30.9 °C for our 3D conical SHiCF‐GO‐PSS evaporator, indicating that the heat loss to the brine and to the environment is small. Under the same experimental conditions, the flat disk evaporator with significantly increased evaporation surface temperature reaches an evaporation rate of ≈1.21 kg m^−2^ h^−1^; whereas, up to 1.69 kg m^−2^ h^−1^ is achieved for our 3D conical SHiCF‐GO‐PSS insulated with a polyethylene foam able to maintain a closer temperature to that of the environment, that is, 30.9 °C.

Next, we look into the different evaporation performance in terms of evaporators’ temperature rise under 15‰ brine over a period of 10 days for the following configurations SHiCF‐GO, SHiCF‐GO‐PSS, SHiCF‐GO‐PSS+air‐laid paper, and SHiCF‐GO‐PSS+air‐laid paper‐PSS, which is represented in Figure 7c. The temperature rise (°C) for the SHiCF‐GO evaporator (blue) remains relatively stable for Day 1 to Day 5, only showing a significant upward trend from Day 6 to Day 10. The temperature increase at the evaporator's interface within 10‐days is direct evidence of the weakening performance of the SHiCF‐GO evaporator in time. In this configuration, salt crystallization ensues, covering a large area of the evaporator's interface, eventually blocking the micro‐/nano‐pores impeding both water supply transport and water vapor diffusion. Impeded water transport and water vapor diffusion hinder evaporation rates and the ability for water to undergo phase change, which causes the evaporation interface temperature rise to increase from Day 1 to Day 10. This is opposite to the temperature rise observed at the SHiCF‐GO‐PSS evaporation interface (yellow), which is maintained within the range of 7.0–7.8 °C over the 10‐days cycle. As earlier conveyed, the PSS functionalization successfully exploits the Donnan equilibrium, limiting the diffusion of Cl^−^ ions into the water supply layer, eventually inhibiting salt crystallization. As a consequence, the vapor and liquid paths are unrestricted and constant evaporation rates and constant evaporative cooling effect ensue (shown in Figure 5c), enabling a constant evaporator's temperature over the 10‐days cycle, with just a slight increase observed at Days 9 and 10, as shown in Figure 7c. This is attributed to the PSS maintaining the physical and chemical properties enabling efficient water transport and evaporation stable in time. When comparing the interface temperature rise of the SHiCF‐GO‐PSS and that of the SHiCF‐GO systems, a lower temperature increase is observed for the PSS functionalized evaporator SHiCF‐GO‐PSS, which is attributed to the continuous high evaporation rates enabled by the constant liquid transport and vapor diffusion in time empowered by the PSS. The timely occurrence of evaporation phase‐change coupled to the effective diffusion of the vapor carries away the heat of vaporization maintaining lower temperatures at the interface and improving energy utilization efficiency.

In the SHiCF‐GO‐PSS+air‐laid paper evaporation system (purple), the excellent capillary effect of the air‐laid paper makes a significant contribution to the continuous water supply at the evaporation interface, significantly improving the evaporation rate between Day 1 and Day 6 with the average interface temperature being reduced by 0.7 °C, when compared to the SHiCF‐GO system. However, due to the continuous evaporation process, the air‐laid paper will be covered by precipitated salt crystals inducing the decay of liquid transport and vapor diffusion in time. Therefore, the interface temperature rise shows a significant upward trend on Day 7 onwards, endowing a rather similar temperature rise performance on Day 10, that is, a temperature rise difference of 0.2 °C, when comparing SHiCF‐GO and SHiCF‐GO‐PSS+air‐laid evaporators. Last, the SHiCF‐GO‐PSS+air‐laid paper‐PSS (green) system, which is the optimum evaporator configuration, shows the most stable temperature rise over time between 7.5 °C and 8.0 °C, that is, no significant increase in time/days, with the lowest of the average temperature rises. Due to the excellent water supply and salt inhibition performance of the polyelectrolyte air‐laid paper, efficient water transport and vapor diffusion effectively prevent salt crystallization, significantly maintaining the overall evaporation performance.

Moreover, as the evaporation efficiency also relies on low temperature increases so to maximize the amount of heat absorbed and invested in evaporation phase‐change, Figure 7d shows the evaporation efficiency under 15‰ brine environment for the different evaporator configurations SHiCF‐GO, SHiCF‐GO‐PSS, SHiCF‐GO‐PSS+air‐laid paper, and SHiCF‐GO‐PSS+air‐laid paper‐PSS. As expected, the evaporator's efficiency exhibits an opposite trend to that of the temperature rise earlier shown in Figure 7c. The accumulation of salt crystals leads to a downward trend in the evaporation rates of SHiCF‐GO and SHiCF‐GO‐PSS wrapped in air‐laid paper, which in turn, increase the evaporator temperature rise; and hence, decrease the evaporator efficiency. The evaporation efficiency of the SHiCF‐GO system wrapped in air‐laid paper within a 10‐days cycle decreases from 89.1% to 86.3%; while, the evaporation efficiency of the SHiCF‐GO‐PSS system decreases from 91.9% to 89.0%. Conversely, the efficiency of the SHiCF‐GO‐PSS and the SHiCF‐GO‐PSS+air‐laid paper‐PSS evaporation systems is maintained within a relatively stable and higher range, with an average evaporation efficiency of 89.7% and 92.3%, respectively. Even in high concentration brine up to 30‰, the SHiCF‐GO‐PSS+air laid paper‐PSS evaporation system can maintain a stable evaporation efficiency of ≈90%, as shown in Figure 7e, confirming the success of the salt resistance capabilities empowered by the PSS polyelectrolytes. The evaporation efficiencies of the evaporator in Hangzhou Bay with a salinity of 9‰, the Yellow Sea with a salinity of 30‰, and the East China Sea with a salinity of 35‰ are 91.5%, 86.9% and 80.4%, respectively, demonstrating the excellent evaporation potential of the our proposed evaporator.

Last, we now compare the PSS functionalized optimum solar evaporator SHiCF‐GO‐PSS and SHiCF‐GO‐PSS+air‐laid paper‐PSS with those of the literature in terms of evaporation rate, evaporation efficiency, brine concentration and test duration, under 1‐Sun irradiation, which are summarized in Table S3 in Section S15 in the Supplementary Information. The SHiCF‐GO‐PSS+air‐laid paper‐PSS achieves one of the highest evaporation rates in 15‰ brine of 1.68 kg m^−2^ h^−1^ compared to 1.38 and 1.60 kg m^−2^ h^−1^ achieved by Chen et al.^[^ ^3^ ^]^ and Li et al.,^[^ ^16^ ^]^ respectively. Up to 1.82 kg m^−2^ h^−1^ has been reported in Alam et al. in a much lower brine concentration of 3.5‰ and reaching higher surface temperatures, these latter conducive to lower evaporation efficiency.^[^ ^25^ ^]^ The 10‐days salt resistance experiment reported in Alam et al., Huang et al., and in our work, provides strong evidence for the long‐term stability in corrosion resistance under higher salinity seawater.^[^ ^7^ ^]^ In the case of Li et al., higher evaporation efficiency but lower evaporation rates of 1.60 kg m^−2^ h^−1^ are reported only within 3 days of operation.^[^ ^16^ ^]^ Such comparison positions our developed SHiCF‐GO‐PSS+air‐laid paper‐PSS evaporator within one of the highest evaporation rates reported for brine concentrations equaling 15‰ with the highest of the evaporation efficiencies over a 10‐days cycle, that is, stable high evaporation rate, high evaporation performance, and efficient energy utilization, over long term operation. A two‐phase area plot representing evaporation rates (kg m^−2^ h^−1^) function of long‐term operation duration (h) for the different investigations has been included in Table S4 in Section S17 in the Supplementary Information, positioning our evaporator within the highest in terms of long‐term operation without requiring service or maintained. We note here that the evaporator with up to 105 h of operation reported in Table S4, Supporting Information, as well as the works by Zhu et al. and Wu et al., reporting up to 144 and 200 h of operation without requiring maintenance, respectively, only consider simulated seawater and no real sea water unlike in the present work.^[^ ^13^, ^27^, ^55^ ^]^ In addition, other evaporator configurations with higher evaporation rate outputs or other PSS functionalization techniques are also suggested as future work. The reasonable design of its functional structure provides new insights for optimizing the in situ interface evaporation performance of solar energy and providing long‐term stable seawater and freshwater extraction equipment.

**Figure 8:**
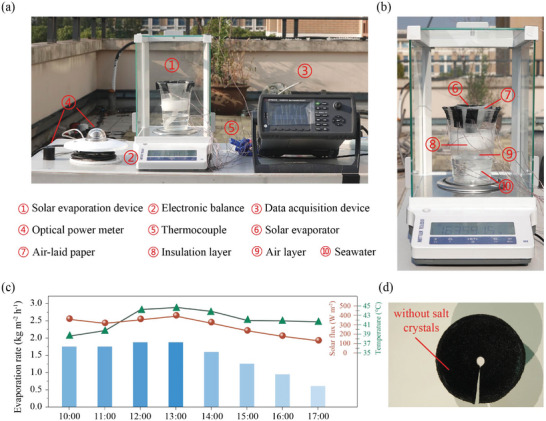
Outdoor experiment of evaporator on roof. a) Outdoors solar evaporator apparatus including SHiCF‐GO‐PSS + air‐laid paper‐PSS evaporator and real seawater reservoir, microbalance, data acquisition device, thermocouples, and optical power meter. b) Snapshot of the microbalance and solar evaporator and real seawater reservoir. c) The evaporation rate (kg m^−2^ h^−1^), solar flux (W m^−2^), and temperature (°C) during the outdoor solar evaporation experiments at different time periods. d) Snapshot of the evaporator after 7 h of experimentation, showing absence of salt precipitation.

Although the laboratory experiment at constant solar flux characterizes the performance of the SHiCF‐GO‐PSS evaporator in a controlled environment, the outdoor realistic weather conditions will inevitably exert dynamic effects on the interface evaporation equipment, such as wind, solar flux, environmental humidity, and environmental temperature fluctuations. In order to further understand the performance of the equipment under these conditions, we conducted an outdoor experiment on a partially sunny day, as shown in **Figure** **8**a,b. The experimental setup was placed on a rooftop platform, using ten thermocouples to characterize the temperature changes of each functional layer and a pyranometer to measure the incident solar flux on the absorber. The total mass loss of seawater from the East China Sea (125°42´, 30°17´) was measured using an electronic balance. The experiment started at 10:00 am and ended around 17:00 pm. The temperature of each stage rose rapidly within the first hour, with the temperature of the solar absorber reaching more than 10 °C higher than the ambient temperature. The solar radiation flux varied significantly within the range of ≈100–500 W m^−2^ due to dispersed cloud layers, resulting in fluctuations in the luminous flux and temperature of the solar absorber as the day progressed. Evaporation rate, solar flux, and temperatures reported can be found in Figure 8c. Despite the lower solar heat fluxes reported along the day during the outdoors experiment, we highlight here the rather high evaporation rates around 1.70 kg m^−2^ h^−1^ between 10:00 am and 13:00 pm, which are equal to those reported throughout laboratory conditions. In the afternoon though, under smaller solar fluxes, the evaporation rate decreased by ½ to 2/3 until the end of the experiment at around 17:00 pm. The reduction on the evaporation rate when compared to laboratory conditions could be attributed to light intensity, temperature, humidity and convection, where typically the outdoors performance was ⅓ of that obtained under controlled conditions in the laboratory. Last, the anti‐salt accumulation ability of the evaporator was not significantly affected, and no salt crystallization was observed on the surface after 7 h of outdoor experiments as shown in Figure 8d, which was in agreement with the absence of salt crystals after 1‐day of operation of our SHiCF‐GO‐PSS+air‐laid paper‐PSS evaporator system in seawater from the East China Sea, as earlier reported in Figure 6g.

The technoeconomic analysis including the cost of raw materials based on different online references as well as equipment and labor cost was assumed as 5–20% of the total price depending on the complexity and difficulty of the fabrication and implementation process having been carried out with specific details and costs provided in Table S5 in Section S18: Cost and cost‐benefit analysis in the Supplementary Information. Overall, the proposed SHiCF‐GO‐PSS+air‐laid paper‐PSS evaporator achieves evaporation cost effectiveness of 2.8 kg h^−1^ $^−1^, which is the third highest amongst the different evaporators analyzed. Only evaporators making use of byproducts or recycling materials such as pomelo peel or red mud have superior cost effectiveness than our SHiCF‐GO‐PSS+air‐laid paper‐PSS evaporator, making it a potential solution for real applications.

## 3 Conclusion

The long‐term operation performance of a 3D inverted conical evaporator subjected to brine has been reported. The proposed evaporator design leverages on the use of a polyanionic electrolyte ionization desalination strategy hindering local salt crystals formation and providing unimpeded continuous water transport and vapor diffusion during solar evaporation operation. Compared to traditional salt resistant techniques, this method not only suppresses the nucleation conditions of salt by precisely regulating ion concentration but also significantly improves the stability and reliability of the interface. By hindering the salt ions diffusion within the water supply path, the SHiCF‐GO‐PSS+air‐laid paper‐PSS evaporator exhibits continuous self‐driving water absorption capacity based on capillary effect without compromising the performance with an average evaporation rate as high as 1.68 kg m^−2^ h^−1^ at over ≈90% efficiency in 15‰ brine, which is maintained within a 10‐days cycle. The proposed configuration is able to maintain a low temperature rise of the evaporation interface and of the bulk water from start to the end with the consequent minimization of heat losses and high evaporation efficiency. This enables continuous water evaporation while at the same time investing evaporative cooling into keeping the evaporator's temperatures constant over time. Moreover, the proposed SHiCF‐GO and wrapped in air‐laid paper functionalized polystyrene sodium sulfonate can achieve high efficacy solar evaporation with a 92% and 90% efficiency in high brine/NaCl concentrations of 15‰ and 30‰ respectively, and in seawater from Hangzhou Bay in the East China Sea with an efficiency of 92% for a salinity of 9‰. Moreover, experiments making use of seawater from the East China Sea with higher salinity of 35‰ and greater number of ions in solution achieved evaporation rates ranging between 1.43 and 1.61 kg m^−2^ h^−1^. In addition, the layer‐by‐layer static deposition methodology adopted anticipated easier and more scalable fabrication procedures, less use of material and chemicals, and further robustness when compared to other strategies such as hydrogels. The evaporator proposed not only provided a new salt resistant solution for the design of solar seawater desalination equipment, ensuring its stability and reliability in practical applications, but also provided an alternative and promising mechanism for the future development of interface solar evaporation driven brine treatment.

## 4 Experimental Section

### Fabrication and Characterization

The schematics of the 3D inverted cone solar evaporator is shown in Figure S1, Supporting Information. The framework of the conical evaporator was made of 130 PPI copper foam (CF) with a porosity of 96.8%, which was soft and fluffy and could be easily bent into a cone with a 45° tip angle.^[^ ^5^, ^14^, ^28^ ^]^ The other relevant dimensions of the cone were an opening diameter of 55.0 mm, a bottom opening of 8.0 mm, and a wall thickness of 2.0 mm. The adopted size was optimized after multiple tests ensuring maximum light absorption; while, providing sufficient water transport. Then, chemical oxidation in alkaline solution treatment was further used to produce uniform copper oxide nano‐blade structured layer decorating the skeleton of the copper foam. Both the micro‐pores and the nano‐structures acted as optical traps, allowing repeated absorption of a large amount of reflected light; while, conferring superhydrophilic wettability.

The chemical oxidation process is shown in Figure S1, Supporting Information.^[^ ^56^ ^]^ First, the copper foam was cleaned in a 2–5 wt% hydrochloric acid HCl solution to remove any oxide layer on the CF skeleton. After being rinsed with distilled water, the CF was immersed into the mixture solution of 2.5 mol L^−1^ NaOH and 0.1 mol L^−1^ (NH_4_)_2_S_2_O_8_ at 70 °C for 30 min to form the superhydrophilic hierarchical micro‐ and/or nano‐structures on the skeleton of the inverted CF cone (c) for the SHiCF evaporator.^[^ ^56^ ^]^ Then, the micro‐/nano‐structured CF or SHiCF was washed again with plenty of distilled water and dried at room temperature until completely dehydrated. Afterward, the prepared superhydrophilic inverted copper foam cone SHiCF was immersed into a 2.5 mg mL^−1^ GO‐water solution in room temperature for 12 h to obtain the black superhydrophilic inverted CF cone decorated with GO or SHiCF‐GO evaporator. The 2.5 mg mL^−1^ GO‐water solution was deemed as the optimum GO concentration as earlier reported.^[^ ^28^ ^]^ After rinsing the evaporator with deionized water and drying it, the SHiCF‐GO evaporator was dip‐coated in a 0.1 g L^−1^ PSS solution to form the polyelectrolyte film layer to obtain the SHiCF‐GO‐PSS, which was subsequently dried overnight.^[^ ^32a^ ^]^ The same procedure was adopted to fabricate the SHiCF‐GO and SHiCF‐GO‐PSS samples for comparison.

Surface microstructure characterization of the porous materials was carried out via scanning electron microscopy (SEM) ZEISS Ultra Plus (Germany). Snapshot of the multi aperture structure of SHiCF in Figure 2d shows the unique skeleton structure with pores in the order of hundreds of micrometers. The light absorption characterization in terms of absorption rates of plane SHiCF‐GO and SHiCF‐GO‐PSS samples was carried out in an ultraviolet–visible–near infrared (UV–VIS–NIR) spectrophotometer PerkinElmer Lamda 950 (America). Moreover, to prove the existence of different interactions between the water and the evaporator configuration as SHiCF‐GO and SHiCF‐GO‐PSS, the types of water molecules absorbed depending on the functional groups were measured by Fourier transform infrared (FTIR) spectroscopy NicoletiN10 MX (America); while, Raman spectra of water confined in SHiCF‐GO and SHiCF‐GO‐PSS were measured using a spectrometer Renishaw inVia Qontor (United Kingdom). The Zeta potential results for SHiCF‐GO and SHiCF‐GO‐PSS were measured in a nano particle zeta potential analyzer Brook Haven Omni (America). The infrared thermal imaging snapshot characterizing the water transport performance of SHiCF‐GO‐PSS samples was taken in an infrared thermal imager FOTRIC 248M‐L29 (China). The actual snapshot representing the water state inside the skeleton was taken by an industrial camera Basler Aca2440‐75uc (Germany). Last, the types and concentrations of ions in seawater were measured using ion chromatograph in a ICS‐5000+/900 (Germany).

### Solar Evaporation Apparatus

To compare the different performance in terms of energy utilization efficiency of the 3D conical evaporator configurations, the evaporation rate and heat transfer performance were conducted. In order to quantitatively analyze and compare the heat transfer performance, a total of nine thermocouples connected to a data acquisition instrument Keysight DAQ970A (America) with an accuracy of ±0.5 °C, and in turn, connected to a PC were utilized as shown in Figure S5, Supporting Information. Solar thermal phase‐change evaporation experiments and measurements of the evaporation rate were carried out by placing the complete solar evaporation apparatus on an electronic scale Mettler Toledo ME204/02 (China) with accuracy of ±0.0001 g so to record the mass change of the bulk brine under 1‐Sun irradiation. The incident power of the simulated sunlight was measured by an optical power meter CEL‐FZ‐A (China). The bulk brine was contained within a clear plastic container of 62.0 mm in diameter and 21.0 mm in height for a total volume of 65.0 mL, with the salinity of 15‰. A Xenon Lamp System CEL‐S500 (China) was employed as the light source to simulate the 1‐Sun irradiation. Comparing the evaporation rate of the pure brine with the experimental data from others in the absence of an evaporator confirmed the accuracy and repeatability of solar heat flux of the light source. To further enhance the transport path supplying water to the evaporator, an air‐laid paper with 0.3 mm in thickness was cut into a fan‐shape and wrapped around the cone. When the lower part of the air‐laid paper was immersed in bulk water, its adhesion to the outer‐side surface of the evaporator was further strengthened through strong capillary force. The conical evaporator wrapped with air‐laid paper‐PSS was then inserted into a concentric circle‐shape polystyrene foam, which was placed at a distance of 20.0 mm from surface level of the bulk brine to prevent conduction heat loss from the evaporator to the bulk brine. The air‐laid paper‐PSS enabled water transport from the bulk water to the superhydrophilic skeleton via capillary effect, also minimizing heat transfer from the evaporator to the bulk. 10‐days cycle solar desalination experiments were then conducted to check the stability of the evaporators.

### Statistical Analysis

Owing to the long‐term duration and operation of the experimental results reported, the results presented in this work did not rely heavily on statistical analysis. Nonetheless for reproducibility, the average and standard deviation of at least three measurements had been carried out for the following results and figures: Zeta potential reported in Figure 2k and evaporation efficiency versus brine concentration in Figure 7e. Two sets of experimental results were used for the calculations of the average and standard deviation reported in height versus time (s) in Figure 2l; and the average and standard deviation of the different measurements taken over a day of evaporation were used in evaporation efficiency versus time (days) in Figure 7d. For these results: 1) Pre‐processing of data: the data was used as is including outliers. 2) Data represented in figures included the mean/average plus/minus the standard deviation (e.g., mean ± SD). 3) The sample size (*n*) for each statistical analysis was at least three independent experimental results. 4) Statistical methods used were only average and standard deviation. 5) Excel or other calculation software were used for the statistical analysis.

For other of the results reported such as some specific experimental characterization results, it was not possible to carry out such statistical analysis and as such individual results or measurements had been reported in the following results and figures: scanning electron microscopy and electron diffraction spectroscopy intensity are shown as single equipment measurements in Figure 2d,f and Figure 2e,g; absorption, transmittance, and intensity are shown as single equipment measurements in Figure 2h–j; mass change in time for the different configurations is shown as single measurement in Figure 3c; snapshots of water fil wetting and wicking, water in pores, and infrared imaging are also single snapshots in Figure 3e–g; experimental concentration function of brine concentration in Figure 4d(I) and function of the concentration of sulfonic acid ions and salt ions in the water supply path in Figure 4f; evaporation rates versus hours and days in Figures 5, 6, 8 or the temperatures in Figure 7a,b,c. Nonetheless, the results reported, for example, in terms of evaporation rate per day showed excellent reproducibility; while, the evaporation rates per hour showed the expected trends.

## Conflict of Interest

The authors declare no conflict of interest.

## Author Contributions

F. Y. Lv contributed to conceptualization and methodology, designed the experimental setup, discussions, writing, reviewing, and editing. J. Miao designed the experimental setup, performed experiment, data curation, discussions, and writing. Z. Y. Wang did the experiment and discussions. J.Hu contributed to supervision and suggestions. D. Orejon contributed to discussions, suggestions, writing, reviewing, and editing.

This work does not require any ethics, patience, or clinical research approval or permission.

## Supporting information



Supplementary Information

Supplemental Video 1

## Data Availability

The data that support the findings of this study are available from the corresponding author upon reasonable request.
