# Supplementary material for: Polyanionic Electrolyte Ionization Desalination Empowers Continuous Solar Evaporation Performance
Source: Adv Mater. 2024 Dec 17;37(6):2410290. doi: 10.1002/adma.202410290 (PMC11817924; doi:10.1002/adma.202410290)
Supplement: Supplementary file 1 — Supplementary Information [file ADMA-37-2410290-s001.docx]

Polyanionic Electrolyte Ionization Desalination Empowers Continuous Solar Evaporation Performance – Supplementary Information

Fengyong Lv ^a^ ^[[1]](#footnote-1)^*, Jie Miao ^a,c^, Zhongyu Wang ^a^, Jing Hu ^b †^, Daniel Orejon ^d,e^ ‡

^a^ School of Urban Construction and Safety Engineering, Shanghai Institute of Technology, Shanghai 201418, China

^b^ School of Perfume and Aroma Technology, Shanghai Institute of Technology, Shanghai 201418, China

^c^ School of Energy and Power Engineering, Dalian University of Technology, Dalian, Liaoning Province, 116024, China

^d^ School of Engineering, Institute for Multiscale Thermofluids, The University of Edinburgh, Edinburgh EH9 3FD, Scotland, UK

^e^ International Institute for Carbon-Neutral Energy Research (WPI-I2CNER), Kyushu University, 744 Motooka, Nishi-ku, Fukuoka 819-0395, Japan

**Abstract**

Solar evaporation contributes to sustainable and environmentally friendly production of fresh water from seawater and wastewater. However, poor salt resistance and high degree of corrosion of traditional evaporators in brine makes their implementation in real applications scarce. To overcome such deficiency, a polyanionic electrolyte functionalization strategy empowering excellent uniform desalination performance over extended periods of time, is exploited. Our 3D superhydrophilic graphene oxide solar evaporator design ensures stable water supply by the enhanced self-driving liquid capillarity and absorption at the evaporation interface as well as efficient vapor diffusion. While the polyanionic electrolyte functionalization implemented via layer-by-layer static deposition of polystyrene sodium sulfonate effectively regulates/minimizes the flux of salt ions by exploiting the Donnan equilibrium effect, which eventually hinders local salt crystallization during long-term operation. Stable evaporation rates in line with the literature of up to 1.68 kg m^-2^ h^-1^ are achieved for up to ten days in brine (15‰ salinity) and for up to three days in seawater from Hangzhou Bay in the East China Sea (9‰ salinity) while maintaining evaporation efficiencies ~90%. This work demonstrates the excellent benefits of polyanionic electrolyte functionalization as salt resistance strategy for the development of high-performance solar powered seawater desalination technology and others.

**Keywords:** Solar interfacial evaporation, Superhydrophilic hierarchical metal copper foam evaporator, Ionization desalination, polyanionic electrolyte functionalization, Donnan equilibrium effect

**SI-1. Cone Evaporator Design**

The 3D inverted conical solar evaporator with a conical top angle of 45° provides enhanced projection area for light incidence while maintaining a reasonable height for conveying water to the conical top. The other relevant dimensions of the cone are an opening diameter of 55.0 mm, a bottom opening of 8.0 mm, and a wall thickness of 2.0 mm. The principle of the solar evaporator studied in this work and the schematic diagram of the 3D inverted cone solar evaporator configuration are shown in Figure S1.

The framework of the conical evaporator is folded into the required cone by 130 PPI copper foam with a porosity of 96.5%. Then chemical oxidation in alkaline solution treatment is used to produce uniform copper oxide nano blade shaped super hydrophilic nanostructure layer, decorate the skeleton surface of copper foam (CF), and change its chemical and physical properties.


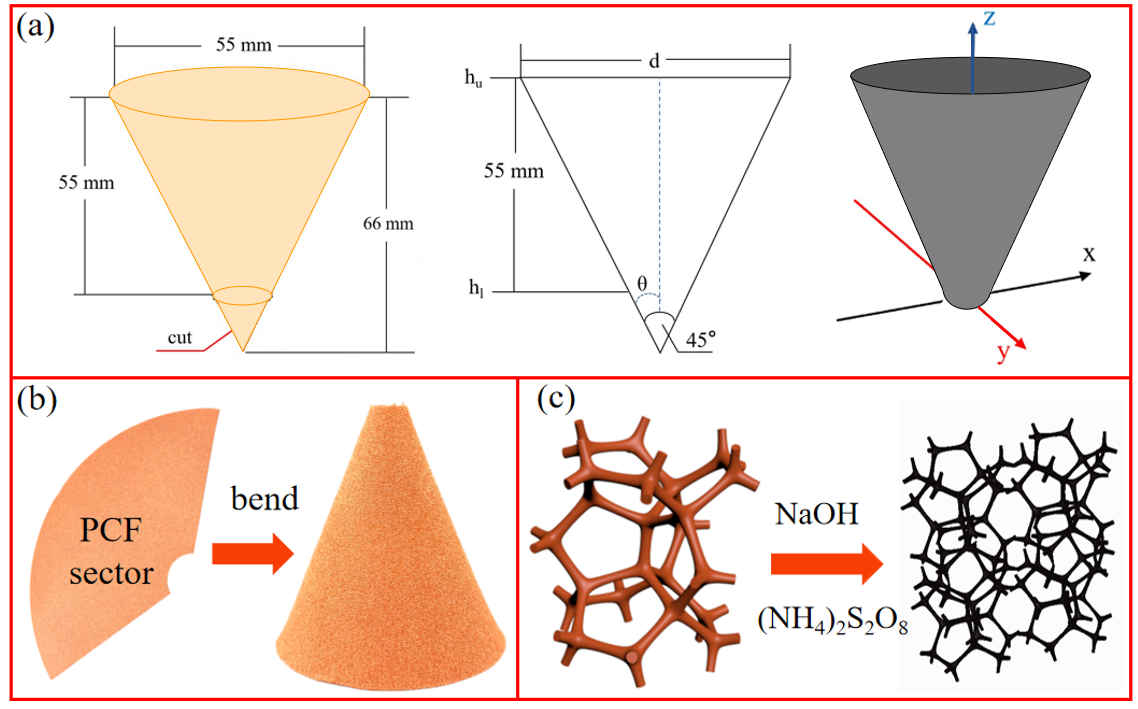


**Figure S1. Cutting and preparation of conical copper foam.** **(a)** Specific dimensions of conical evaporator. **(b)** Bending process of cavity structure. **(c)** Black oxide layer is produced through chemical oxidation.

**SI-2. Fourier transform infrared FTIR of SHiCF-GO evaporator**

Fourier transform infrared (FTIR) spectroscopy was used to identify the functional groups of SHiCF-GO and SHiCF-GO-PSS solar evaporators with polyelectrolyte coating (Fig. 2i). FTIR spectrum for SHiCF-GO in Fig. S2 shows different peaks with centers at 1257 and 1731 cm^-1^ corresponding to C−O−C and C=O bonds, respectively, which prove the existence of a large number of oxygen-containing functional groups.^[1]^ When comparing the FTIR spectral vibration of SHiCF-GO in Fig. S2 with the spectral vibration band of SHiCF-GO-PSS in Fig. 2i, the spectral peaks at 822 cm^-1^ and 906 cm^-1^ are related to the S-O stretching vibrations and the peaks at 1100 cm^-1^ , at 1180 cm^-1^ and at 1370 cm^-1^ are related to the S=O stretching vibrations, which confirm the introduction and presence of sulfonic acid groups.^[2]^

**Figure S2. FTIR diagram of SHiCF-GO.**

**SI-3. Energy Dispersive X-ray spectroscopy EDS of SHiCF-GO evaporator**

By detecting the energy dispersive X-ray spectroscopy (EDS) of the surface of the SHiCF-GO-PSS evaporator, it was confirmed that the pores of the irregular network skeleton structure were filled with sodium polystyrene sulfonate. EDS spectroscopy detected the presence of Na element, confirming the presence of a large amount of -SO_3_Na connected to the mesoporous network.

**
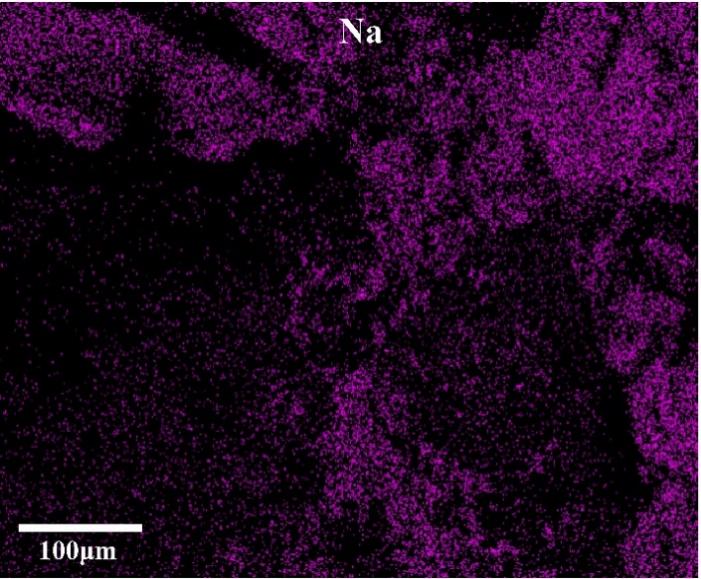
**

**Figure S3. EDS of Na element on the surface of SHiCF-GO-PSS evaporator.**

**SI-4. Salt crystallization behavior of conical copper foam and air-laid paper.**

In the SHiCF-GO evaporation system, the cone evaporator is wrapped in air-laid paper and its bottom is immersed in simulated seawater. The top of the inverted cone is the end of water transportation, where the salt concentration first reaches saturation, leading to the first appearance of a salt ring at the top and continuously spreading downwards.^[3]^ However, as a water supply layer, air-laid paper can also become the object of salt crystal adhesion. Salt crystals adhere to the interface between the air-laid paper and the inverted cone, seriously hindering the evaporation performance. The salt crystals attached to the outer wall of copper foam are due to the adhesion of air-laid paper, which shows the necessity of air-laid paper modification. The PCF evaporator wrapped in air-laid paper also exhibits a similar phenomenon of salt precipitation, as shown in the Fig. S3. However, the low water delivery capacity of PCF makes the upward water supply insufficient, which leads to most salt crystals are separated from the air-laid paper and adhered to the outer wall of copper foam.


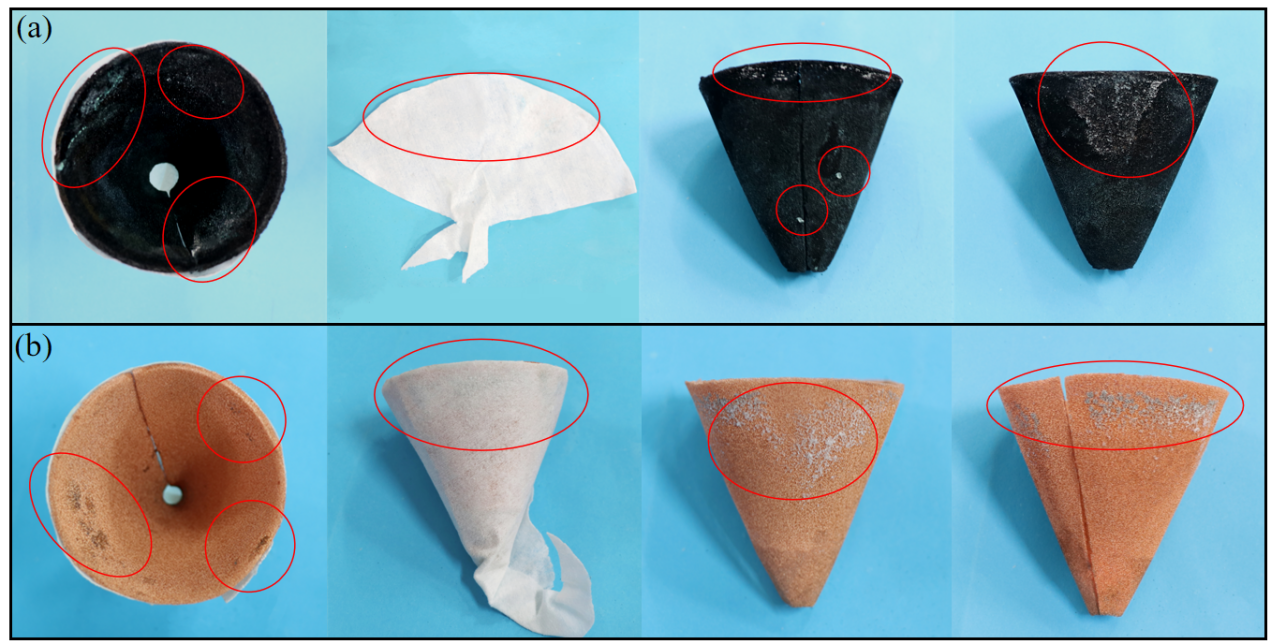


**Figure S4. Salt crystallization behavior:** **(a)** air-laid paper coated SHiCF-GO and (b) conical pristine copper foam PCF.

**SI-5. Solar Evaporator Experimental Apparatus**

The evaporation system consists of a data acquisition instrument Keysight DAQ970A (USA) with an accuracy of ± 0.5 ℃, a PC, a thermocouple, an electronic scale Shimadzu AUW120D (Japan) with an accuracy of ± 0.0001g, and a xenon lamp system light source with a CEL-S500 (China).

**
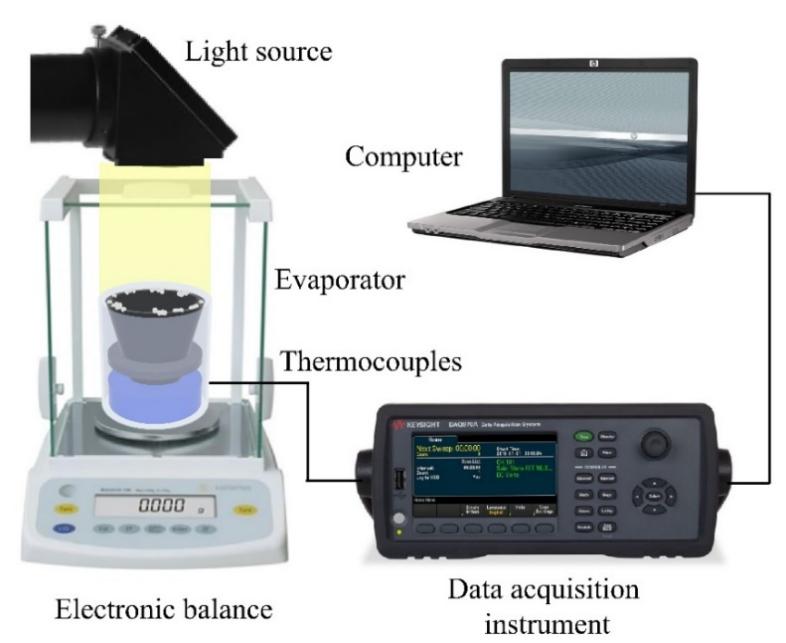
**

**Figure S5. Experimental apparatus.**

**SI-6. Calculation formula based on Donnan equilibrium**

Modeled by the boundary-layer differential equation for mass transfer, the local salt concentration (Na) can be quantificationally described as following:^[4]^

$C_{a}=C_{t}exp\left(\frac{J_{w}}{\kappa}\right)$ (S1)

where *J*_w_ refers to the water evaporation rate, *κ* is the diffusion rate of the salt ions in the water supply layer, *C*_t_ is the concentration of the salt ions diffusing into the water supply layer. *C*_t_ is approximately equal to the concentration of bulk brine (*C*_b_, such as NaCl). Therefore, it will be an ideal strategy to prevent salt from accumulating on the surface of the solar absorber by reducing the number of salt ions entering into the water supply layer.^[4]^

 (S2)

There is a well-known Donnan effect, in which because of the fixed charge (anions, such as COO−) in the water supply layer, counter-ions (cations, such as Na^+^) are confined within the water supply layer due to electric neutrality. These confined counter-ions create a high chemical potential and alter the salt ions partition equilibrium between the water supply layer and bulk brine. Base on the thermodynamics, a new partition equilibrium is established as follows:^[4-5]^

**$C_{t}\left(\varphi+C_{t}\right)=C_{b}^{2}$** (S3)

where *φ* is the concentration of the confined anion (such as HSO_3_^-^) and counterions (such as Na^+^) in the water transport path; *C_t_* refers to the ion (Na^+^, Cl^-^) concentration that diffuses into the water transport path; *C_b_* is salt concentration in simulated seawater. Consequently, the amount of the salt ions diffusing into the water supply path (*C_t_*) can be regulated by the concentration of the confined counter-ions.

The weighted average absorption rate of the evaporation interface within the full spectral range is calculated according to the following formula:^[6]^

$\bar{\varepsilon}_{ave}=\bar{\varepsilon}_{u}\times k_{u}+\bar{\varepsilon}_{v}\times k_{v}+\bar{\varepsilon}_{i}\times k_{i}$ (S4)

where $\bar{\varepsilon}_{ave}$ is the weighted average absorption rate for the whole spectrum of incident light, $\bar{\varepsilon}_{u}$ is the spectral absorption rate of the surface for ultraviolet light (250 nm - 390 nm), $\bar{\varepsilon}_{v}$ is the spectral absorption rate of the surface in visible light (390 nm - 770 nm), and $\bar{\varepsilon}_{i}$ is the spectral absorption rate of the surface for infrared light (770 nm - 2500 nm), while *k_u_* is the proportion of ultraviolet light energy, *k_v_* of visible light energy and *k_i_* of infrared ray energy.^[6]^ The proportions of ultraviolet light energy, visible light energy and infrared ray energy in total solar energy are 3%, 45% and 52%, respectively.^[7]^ The weighted average absorption rate of SHiCF-GO-2.5 in the entire spectrum calculated by the following formula is 93.4%, while the weighted average absorption rate of SHiCF-GO-2.5-PSS in the entire spectrum is 93.2%, indicating that the addition of PSS has no significant effect on the absorption performance of the evaporation surface.

The formula for calculating the incident simulated sunlight is expressed as:^[8]^

$P_{in}=\int_{L}^{L+H} 2\pi\left[R-\left(h-L\right)tan\theta\right]\bar{\varepsilon}_{ave}I tan\left(\theta\right)d\left(h\right)$ (S5)

where *P_in_* is the power of the incident simulated sunlight, *L* is the distance from the light source to the top of the conical evaporator (*L* = 10 cm), *H* is the vertical height of the top to the bottom of the cone evaporator (*H* = 5.5 cm), *R* refers to radius of cross section at the top of conical evaporator (*R*=2.75 cm), $\bar{\varepsilon}_{ave}$ is the weighted average absorption rate of incident light, *h* refers to the vertical height from a certain position of the conical interface to the light source, *I* refers to the intensity of incident light inside the conical evaporator after fitting, which will decay with the increase of distance from the light source, *θ* represents that half of the apex angle is 22.5° which is formed by the bus bar of the cone-shape evaporator and the vertical line, the related dimensional parameters of inverted cone evaporator are shown in Fig. S1.

The formula for calculating the integrated average light intensity is as follows:^[9]^

$I_{ave}=\frac{P_{in}}{Asin\left(\theta\right)}$ (S6)

where *I_ave_* refers to the integrated average light intensity of the incident light on the inner surface of the conical evaporator, *A* is the inner surface area of the conical evaporator.

The evaporation rate is calculated by normalizing the projected area of the cone as follows:^[10]^

$e=\frac{\Delta m}{\tau A_{pro}}$ (S7)

where *e* is the evaporation rate per unit area and per unit incident light intensity, *A_pro_* refers to the projected area of the conical evaporator,^[8, 10a]^ *Δm* is the reduced net mass of bulk water under 1-Sun irradiation and *τ* is the evaluated evaporation time at steady state.

While the evaporation efficiency or the photothermal conversion efficiency *η* is estimated as follows:^[8, 10a]^

$\eta=\frac{\Delta mh_{fg}}{\tau P_{in}}$ (S8)

where *h_fg_* represents the total enthalpy change of water including latent heat. All efficiencies are calculated by subtracting the evaporation rate in the dark environments.

**SI-7. Stability testing of polyelectrolyte PSS**

The SHiCF-GO samples deposited with PSS, SHiCF-GO-PSS, were immersed in deionized water for 9 hours. As shown in Figure S6, there was no significant discoloration of the water system and the sample morphology remained unchanged.


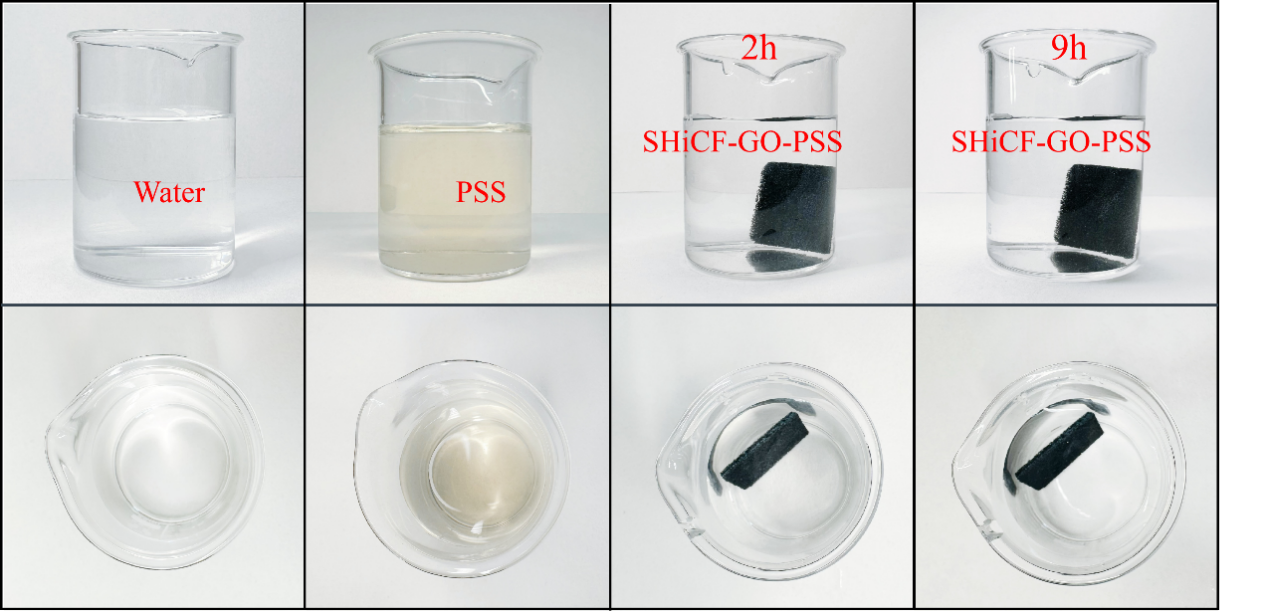


**Figure S6.** **PSS and GO stability testing.**

**SI-8. Salt crystallization behavior of evaporators with different configurations**

Local crystallization is a positive strategy in evaporator design to continuously generate steam and simultaneously harvest salt. The water delivery channel with n-shaped cross section is made of air-laid paper, which is covered on the surface of polystyrene foam and bent downward to form a frustum with skirt as shown in Fig. S7. The bulk brine is transported upward from the skirt to the table top of the air-laid paper circular truncated cone, and then the super hydrophilic copper foam is wetted. The water supply layer with T-shaped cross section is made by threading the central part of the air-laid paper through the center of the polystyrene foam ring and sinking downward to form an umbrella structure. The bulk brine is transported from the umbrella handle upward to the air-laid paper umbrella surface, and finally wet the foam with the skeleton. Capillary force drives the water flow to transfer ions from the bulk liquid. As the water in the evaporation area gradually decreases, the increase in solution concentration will cause the ions carried to transform into salt crystals near the end. By carefully controlling the flow direction and evaporation area of the water supply, salt crystallization can be limited to specific local areas that do not affect evaporation.


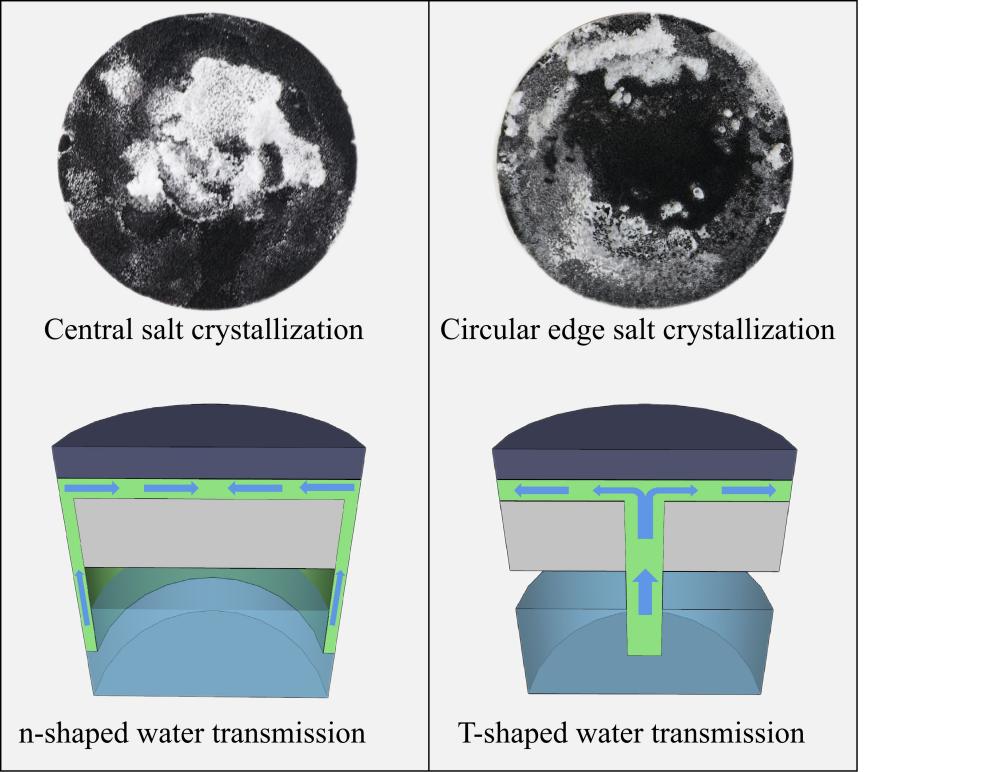


**Figure S7. Types of water transport paths and phenomena of salt precipitation.**

**SI-9. Air-laid paper and pore dual channel water supply**

In the solar evaporation experiments, the SHiCF-GO is tightly adhered to the air-laid paper, which is in turn in direct contact with the bulk water. Hence, the SHiCF-GO outermost interface is just wetted by the water absorbed from the air-laid paper forming a water layer over the GO instead of at the SHiCF-GO interface, which ensures that the surface morphologies and chemical properties of the solar evaporator and those of the SHiCF-GO interface remains unchanged. Within nine hours of a solar evaporation experiment, the evaporation interface remains wet as a consequence of the excellent water absorption and transport characteristics owed to the synergistic cooperation of SHiCF-GO continuously wetted by the air-laid paper. As water turns into vapor, water molecules wick upwards and towards the evaporating interface along the superhydrophilic skeletons of the SHiCF-GO filling the nanoscale vacancies between the blade-like nanostructures around the skeleton. Unlike ordinary solar evaporators where the vapor diffusion mechanism occurs from the interface of the water bulk towards the air, in the presence thermal localized evaporation within our 3D conical evaporator, the vapor diffusion mechanism follows two distinctive vapor diffusion paths, namely the outside surface of the air-laid paper and the interconnected pores of the SHiCF-GO. The stacked skeleton of the CF provides a path for the vapor diffusion via the different adjacent interconnected pores. When looking at the system in terms of energy and heat transfer, upon the incidence of the simulated sunlight at 1-Sun, the incident light is converted into heat by solar-thermal conversion. Heat is then absorbed by the SHiCF-GO and conducted across the CF towards the air-laid paper-SHiCF-GO interface and towards air-laid paper-air interface. Water infiltrated within the nanoscale pores between the blade-like structures of the SHiCF-GO and the air-laid paper then heats up generating water vapor. The vapor then diffuses through the pores from the inner wall of the SHiCF-GO towards the ambient as well as from the air-laid paper towards the ambient.


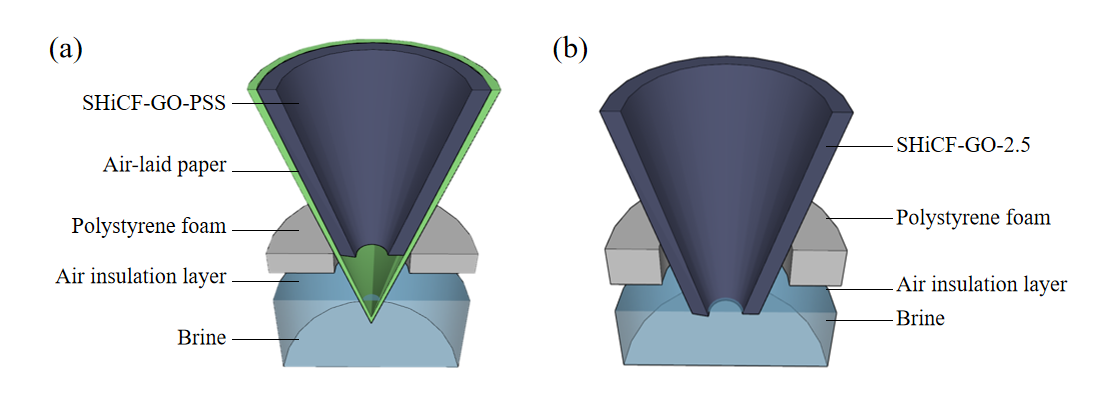


**Figure S8.** **The contribution of air-laid paper to water transportation. (a)** The cone wrapped in air-laid paper serves as the evaporation system for the evaporator. **(b)** Cone evaporator without air-laid paper wrapping.

**SI-10. A small amount of salt reflux can achieve self-cleaning**

The evaporation of the conical evaporator causes salt crystals to accumulate at the top, and when it is left standing in a dark environment, the solar driven interface evaporation process stops. However, under dark conditions, the capillary effect still drives the skeleton to pump brine to keep the skeleton moist, causing the top salt crystals to slowly dissolve into the water film, increasing the concentration of the salt crystallization site, and ultimately reflux back into the bulk brine to achieve self-cleaning.

**
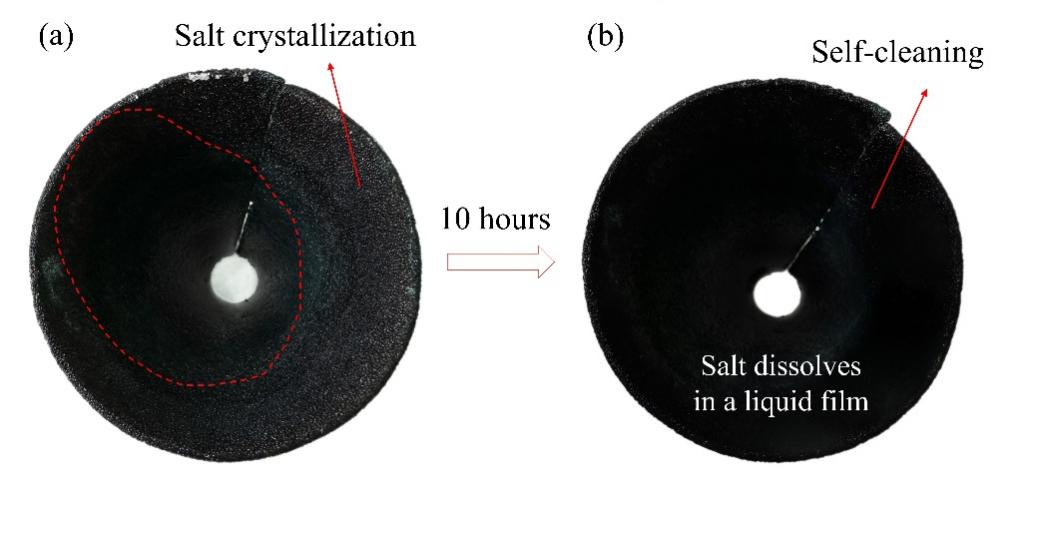
**

**Figure S9. Schematic diagram of cone salt crystallization in dark conditions.**

**SI-11. Accurate measurement method for evaporator quality in dry state**

The evaporator in a moist state is placed under sunlight for evaporation and drying, and the mass can be measured when it stabilizes.

**Figure S10. Mass reduction data of** liquid film in SHiCF-GO and SHiCF-GO-PSS evaporation systems.

**SI-12. Verify the Donnan balance measurement method**

If the local salt concentration around the solar absorber increases during the water evaporation process, salt crystallization will inevitably occur. Therefore, it is important and may be desirable to adjust the salt concentration in the water supply path. The ion balance formula that depends on the contribution of the Donnan effect, in which the cation (such as Na^+^) is limited in the water transport layer in order to keep the solution neutral due to the additional anion in the water transport path. These restricted cations produce high chemical potential, thus changing the salt ion distribution balance between the water supply path and a large amount of brine. Using the Donnan effect, researchers have developed a series of solar evaporators based on hydrogel, whose unique feature is the negative charge fixed on the structural wall, making it salt resistant.

The dry SHiCF-GO cone was immersed in PSS solution for 30 minutes and placed on an electronic balance, which was simulated under sunlight to detect mass reduction data. When the mass approached a plateau, the mass of PSS deposited in the cone can be accurately calculated, which can then be used to calculate *φ*. The dried SHiCF-GO-PSS was wrapped in air-laid paper and its bottom end was immersed in NaCl simulated seawater to simulate the establishment process of Donnan equilibrium. Afterwards, the cone of SHiCF-GO-PSS was taken out and placed on an electronic balance, and it was left standing under simulated sunlight to detect data reduction in quality. When the mass approaches a plateau, the mass of NaCl deposited in the cone can be accurately calculated, and then *C*_t_ can be calculated.

**SI-13. Wettability and Capillary Raising Characterization**

In order to measure the wettability of SHiCF-GO and SHiCF-GO-PSS, inverted conical samples with the same size were prepared using the above preparation method. Evaluate the permeability of SHiCF-GO and SHiCF-GO-PSS by conducting capillary rise characterization experiments on different surfaces. In the capillary rise experiment, the sample is suspended 50 mm above the simulated seawater, and the water level is slowly raised through the lift table until the water level is just below the bottom of the copper foam cone sample.^[13]^ Water is absorbed from bulk water by the capillary channel of air-laid paper and transported to skeleton of the SHiCF-GO-PSS. Due to the skeleton not being in direct contact with bulk seawater, the snapshot shows the hollow state of the pores, proving the successful formation of the ultra-thin water layer.


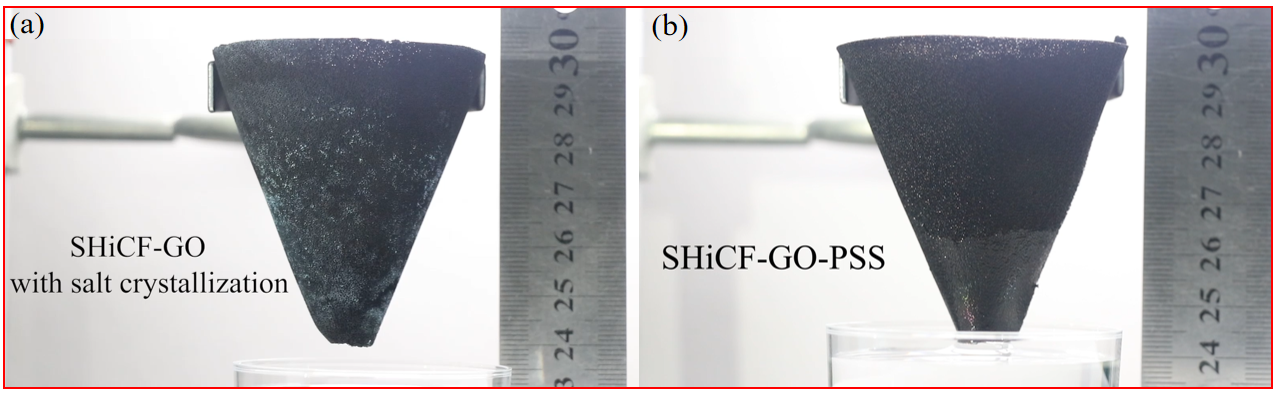


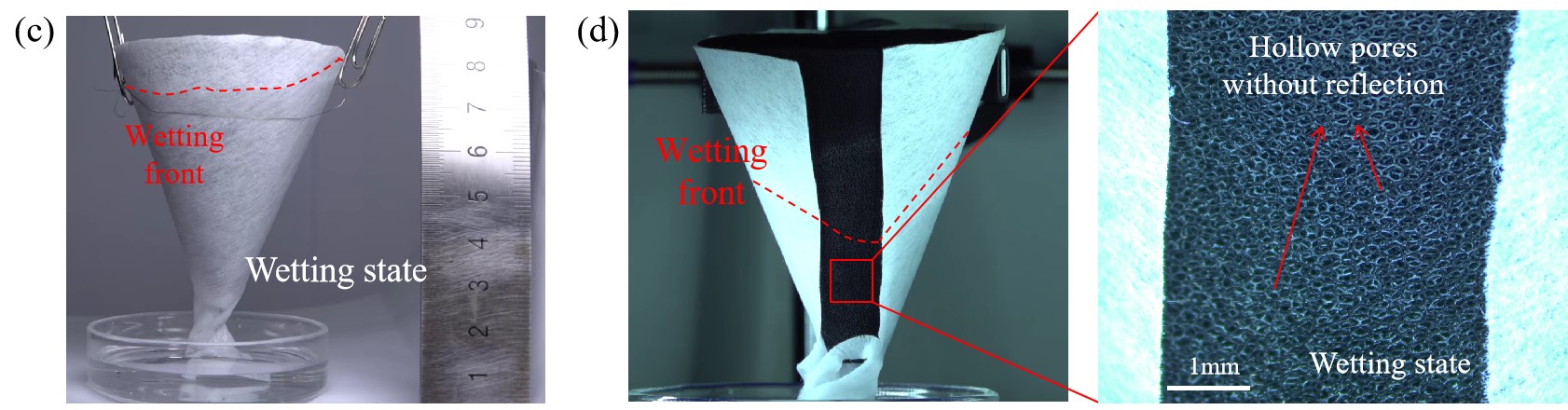


**Figure S11.** **Capillary characterization experiment: (a)** SHiCF-GO and **(b)** SHiCF-GO-PSS. **(c)** Actual snapshot of the wetting front spread of the evaporator wrapped in air-laid paper. **(d)** Actual snapshot of seawater state in the evaporator skeleton wrapped in air-laid paper.

**SI-14. Evaporation rate and efficiency of evaporation systems with different configurations**

Multiple evaporation experiments were conducted to verify the importance of each structure. Due to the presence of sulfonic acid groups, Cl^-^ is prevented from immersing in the water supply layer, and the crystallization salt in the evaporator with PSS is inhibited, indicating that the polyelectrolyte has good water purification durability under high salt environment conditions.

**Table S1.** **The evaporation rate and dark evaporation rate of different configurations of evaporation systems.**

| Evaporation system | Evaporation rate on the 1st day  (kg m^-2^ h^-1^) | Evaporation rate on the 10th day  (kg m^-2^ h^-1^) | Dark evaporation rate  (kg m^-2^ h^-1^) |
| --- | --- | --- | --- |
| Brine | 0.13 | ---- | 0.07 |
| SHiCF | 1.14 | 0.87 | 0.11 |
| SHiCF-GO | 1.39 | 0.94 | 0.12 |
| SHiCF-GO-PSS | 1.44 | 1.42 | 0.09 |
| SHiCF-GO+air-laid paper | 1.67 | 1.35 | 0.08 |
| SHiCF-GO-PSS+air-laid paper | 1.68 | 1.62 | 0.09 |
| SHiCF-GO-PSS+air-laid paper-PSS | 1.68 | 1.67 | 0.08 |

**Table S2.** **The evaporation efficiency of SHiCF-GO-PSS evaporation systems.**

| Salinity(‰) | Radiation(%) | Convection(%) | Conduction(%) | Evaporation efficiency  (%) |
| --- | --- | --- | --- | --- |
| 15 | 4.7 | 3.0 | 1.2 | 91.1 |

**SI-15. Compared to other works**

Compared with the recently reported solar evaporation system, the evaporator with PSS barrier reported in this work has medium to high level evaporation performance and durability under 1 solar irradiation.

**Table S3.** **The evaporation rate and dark evaporation rate of different configurations of evaporation systems.**

| Author | Evaporator | Evaporation rate  (kg m^-2^ h^-1^) | Evaporation efficiency  (%) | Salinity  (‰) | Durability test |
| --- | --- | --- | --- | --- | --- |
| Alam. et al^[14]^ | Cellulose Composite Aerogels | 1.81 | 92.5% | 3.5 | >10 h |
| Chen. et al^[15]^ | Self-Recovering Hydrophobicity | 1.38 | 86.9% | 15 | >90 d |
| Deng. et al^[16]^ | U-shaped hydrogel tubes | 1.31 | ---- | 25 | >7 d |
| Huang. et al^[17]^ | Melamine@Silicone nanoparticles | 1.65 | ---- | 10 | >10 h |
| Li. et al^[18]^ | Tree root-based evaporator | 1.60 | 96.5% | 15 | >3 d |
| Our work | **SHiCF-GO-PSS** | **1.68** | **92.3%** | **15** | **10 d** |

**SI-16. Interface evaporation experiment based on seawater**

After settling for a period of time, the seawater appears clear and transparent for use in solar interface evaporation experiments as shown in Figure S12 while the concentrations of the different types of ions present in the seawater from the Hangzhou Bay in the East China Sea, the Yellow Sea and the East China Sea are shown in Figure 6d. It can be seen that the concentration of Mg^2+^ ions is only 11% of the concentration of Na^+^ ions, while the concentration of anions such as F^-^, Br^-^, NO_3_^-^ and PO_4_^3-^ ions is quite small. When looking into the different seawater samples, no obvious salt crystallization is observed after 3 days of continuous operation making use of seawater from the Hangzhou Bay with a salinity of 9‰ and from the Yellow Sea with a salinity of 30‰. Further in the case of sweater from the Hangzhou Bay no major changes in the evaporation rates are observed after 3 days of continuous operation with values between 1.63 kg m^-2^ h^-1^ and 1.68 kg m^-2^ h^-1^, whereas in the case of the seawater from the Yellow Sea a decrease on the evaporation rate is reported, which is attributed to the increase in ion concentration of most elements causing a greater burden on the anti-salt accumulation system of the evaporator when compared to the composition of the seawater from the Hangzhou Bay. Such challenge may be solved by regulating further the specific ion concentration of the polyelectrolyte by making use of different polyelectrolytes with higher ionic strength or increasing the polyelectrolyte density. Last, the evaporator performance from seawater from the East China Sea sees salt crystallization daily presumably as a consequence of the high salinity content of 35‰, which is more than 3-fold that of Hangzhou Bay and 2-fold that of the Yellow Sea. Under this seawater evaporation experiments, the evaporation rate ranges between 1.43 kg m^-2^ h^-1^ and 1.61 kg m^-2^ h^-1^, and although it decays daily it does further recover over night with starting evaporation rates at around 1.60 kg m^-2^ h^-1^.


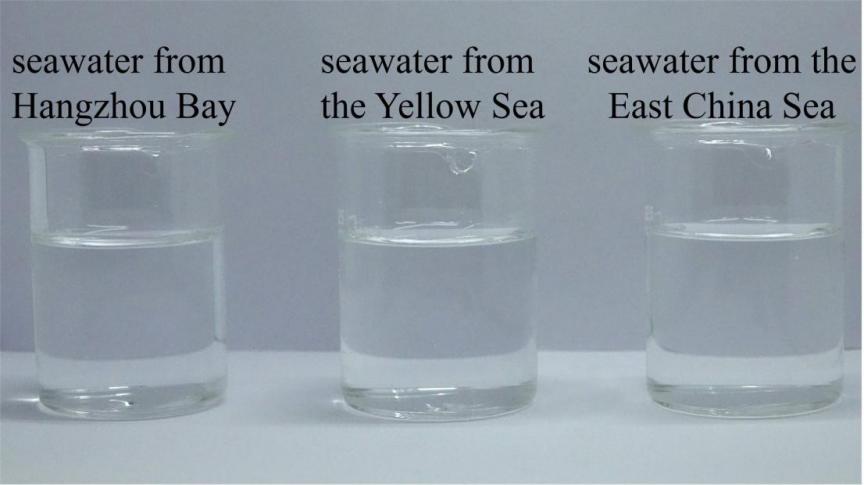


**Figure S12. Photos of seawater after settling for a certain period of time.**

**SI-17. Comparison of evaporation performance of reported solar evaporators**

In order to maximize the thermal utilization efficiency of the vaporization system, researchers mainly increase the heat generation rate by improving light absorption, and minimize heat loss by controlling heat conduction, convection, and radiation. In fact, most photothermal materials have significant photothermal conversion performance. Common photothermal conversion materials, such as gold nanoparticles, plasmonic metals, ionic semiconductors, and carbon-based materials, convert almost all absorbed light into heat with a light absorption rate exceeding 90%. Carbon based materials and conjugated polymers are more suitable for broadband light absorption due to their conjugated structures. So far, carbon-based materials such as carbon black, graphene, graphene oxide, reduced graphene oxide, and polypyrrole have been widely used in the preparation of evaporators. We choose graphene oxide modified porous copper foam as the light absorption layer, which increases the light absorption rate to 94%, which is at a medium high level compared with the existing light absorption materials.

The water supply process within the interface evaporation is a key factor affecting the evaporation rate and salt tolerance sustainability. Although 1D, 2D, and 3D waterway designs ensure sufficient water supply, the abundant water in the materials hinders steam diffusion and heat localization. In addition, the strategy of relying on waterway design to achieve seawater desalination limits the end of water transportation. The salt ions at the end of the waterway are most prone to saturation, which inevitably leads to salt crystallization. This is a key challenge that salt resistance cannot overcome. Based on the design of a 3D water conveyance channel, we use air-laid paper to achieve thin film water conveyance, ensuring sufficient water supply while effectively utilizing heat.

In recent years, three innovative methods have been proposed successively. The first method is to use diffusion and convection to dissolve salt ions from the evaporating surface into the water below. The second method is to achieve desalination and salt collection through structural design. The third method is to use the oxygen-containing functional groups of polyelectrolytes to achieve ionization equilibrium and suppress salt saturation. We utilized the Donnan effect induced by sulfonic acid groups obtained from the hydrolysis of sodium polystyrene sulfonate, which fundamentally inhibited salt nucleation without sacrificing insulation performance and water supply rate. Compared with ordinary salt resistant evaporators, we have extended the operating time of the evaporator to 90 hours, which is at an advanced level.

Table S4 Comparison of evaporation performance of reported solar evaporators.

| **Material** | **Solar absorbance** | **Water supply mode** | **Evaporation rate**  **(kg m^-2^ h^-1^)** | **Anti salt accumulation mechanism** | **Durability:**  **evaporation time/run time (h/h)** | **Ref** |
| --- | --- | --- | --- | --- | --- | --- |
| Silver(Ag)NP/carboncloth | 92.39% | Self absorbing water | 1.36 |  |  | 19 |
| BlackAg/lterpaper | 92% | 2D | 1.28 | Diffusion backflow | 40/40 | 20 |
| Tifoam | 97% | 2D | 1.79 | Janus structure | 20/20 | 21 |
| Carbonblack(CB)NPs/ cellulose | 96% | 1D | 1.62 | Janus structure | 9/9 | 22 |
| Graphenesponge/graphene foil | 97.4% | 1D | 2.01 |  | 25/25 | 23 |
| PDA/PEI/PPy/polyamide | 93% | 2D | 1.43 | Diffusion backflow | 30/30 | 24 |
| Ag/poly(sodium-p styrenesulfonate)/agarosegel | 93.14% | Thermal insulation water supply | 2.10 | Marangoni effect | 24/24 | 25 |
| Fe_3_O_4_/carbon | 99% | 1D | 1.32 |  |  | 26 |
| GO/ filter paper | 95% | 1D | 1.26 | Janus structure | 24/24 | 27 |
| PPy/air-laidpaper | 99% | Thermal insulation water supply | 1.38 |  |  | 28 |
| Polyvinylalcohol(PVA)/Ti_2_O_3_ | 96% | Self absorbing water | 3.6 | Marangoni effect | 30/72 | 29 |
| PPy/PVA | 95% | Self absorbing water | 3.2 |  |  | 30 |
| Chitosan aerogel | 96% | Self absorbing water | 1.76 | Diffusion backflow | 24/24 | 31 |
| Chitosan/Bamboo fiber/Biochar | 90% | 2D | 1.52 | Diffusion backflow | 40/40 | 32 |
| Ag NPs/PDA/Wood | 96% | 2D | 1.58 | Diffusion backflow | 25/25 | 33 |
| Ppy/PDA/Sponge | 97% | 2D | 1.7 | Marangoni convection | 24/24 | 34 |
| CNTs/SiO_2_ nanofiber | 98% | 2D | 1.53 | Interactive macroporous/diffusion backflow | 20/20 | 35 |
| rGO/Chitosan/Fabric | 97.2% | 1D | 2.02 | Marangoni effect | 9/9 | 36 |
| Photosensitive resin/CNTs | 95% | 2D | 1.88 | Marangoni effect | 84/168 | 37 |
| SiO_2_/Glass ball/Epoxy resin |  | 1D | 1.33 | Janus structure | 50/50 | 38 |
| **Material** | **Solar absorbance** | **Water supply mode** | **Evaporation rate**  **(kg m^-2^ h^-1^)** | **Anti salt accumulation mechanism** | **Durability**  **evaporation time/run time (h)** | **Ref** |
| Al–AlNxOy | 94.5% | 1D | 1.707 | Janus structure | 105/105 | 39 |
| PAAS/Biomass | 98% | 2D | 1.83 | Donnan effect | 70/70 | 40 |
| Graphene/alginate | 95% | 2D | 1.45 | High osmotic pressure and ion rejection | 60/60 | 41 |
| Wood/ plasma metal | 99% | Self absorbing water | 11.8 (10-sun) | Diffusion backflow | 48/144 | 42 |
| Poly (N-isopropylacrylamide)  /carbon nanotubes | 90% | 3D | 3.3 | Diffusion backflow | 200/200 | 43 |
| **Copper foam/GO/PSS** | **93.6%** | **3D water film** | **1.71** | **Donnan effect** | **90/90** | **Our work** |
| **Average** | **95.2 ± 2.5** |  | **1.83 ± 0.62** |  | **40 ± 27** |  |

**SI-18.** **Cost and cost-benefit analysis**

We have analyzed the cost and cost-benefit of this work. Table S5 shows that the SHiCF-GO-PSS solar evaporator can provide 90 hours of stable operation at lower cost and higher cost-benefit, showing the potential of practical application. The total cost includes not only the materials used, but also the use and consumption of equipment and manpower during the experimental process. The estimated individual cost of the materials was based on references from Limited Company (China), Aladdin Biochemical Technology Co., Ltd. (China), China National Pharmaceutical Group Chemical Reagent Co., Ltd. (People's Republic of China), and Alibaba (China). The assumed equipment cost (CE) and labor cost (CM) are 5% -20% of the total price, depending on the complexity and difficulty of the experimental process.

**Table S5.** **Evaporation performance, durability, and costs of various solar evaporators.**

| **Raw material prices and cost of equipment (C_E_) and manpower (C_M_)** | **Estimated single item cost**  **($/1*1*0.01 m^-3^)** | **Total cost**  **($/1*1*0.01 m^-3^)** | **Evaporation rate**  **(kg m^-2^ h^-1^)** | **Cost-effectiveness**  **(kg h^-1^ $^-1^)** | **Durability: Pure evaporation time (h)** | **Ref.** |
| --- | --- | --- | --- | --- | --- | --- |
| **Ammonium molybdate** | $7.61 | **100.63** | **2.19** | **2.2** | **20** | **42** |
| Glucose | $1.09 |  |  |  |  |  |
| Chitosan | $56.58 |  |  |  |  |  |
| Soluble starch | $28.29 |  |  |  |  |  |
| Extra C_E_ & C_M_ | $10.06 (10%) |  |  |  |  |  |
| **Polyvinyl alcohol** | $2.19 | **47.33** | **2.185** | **4.6** | **20** | **43** |
| Chitosan | $38.25 |  |  |  |  |  |
| Glutaraldehyde | $0.16 |  |  |  |  |  |
| Red mud | $2 |  |  |  |  |  |
| Extra C_E_ & C_M_ | $4.73 (10%) |  |  |  |  |  |
| **Ti_3_AlC_2_ MAX** | $432.76 | **566.78** | **2.08** | **0.4** | **20** | **44** |
| Dopamine hydrochloride | $9.89 |  |  |  |  |  |
| Lithium fluoride | $1.72 |  |  |  |  |  |
| Sodium chlorite | $4.32 |  |  |  |  |  |
| Balsa wood | $33.07 |  |  |  |  |  |
| Extra C_E_ & C_M_ | $85.02 (15%) |  |  |  |  |  |
|  |  |  |  |  |  |  |
|  |  |  |  |  |  |  |
| **Raw material prices and cost of equipment (C_E_) and manpower (C_M_)** | **Estimated single item cost ($/1*1*0.01 m^-3^)** | **Total cost ($/1*1*0.01 m^-3^)** | **Evaporation rate (kg m^-2^ h^-1^)** | **Cost-effectiveness (kg h^-1^ $^-1^)** | **Durability (h)** | **Ref.** |
| **Pine block** | $23.18 | **418.79** | **2.07** | **0.5** | **10** | **45** |
| Sodium dicyanamide | $45.89 |  |  |  |  |  |
| Pyrazine | $49.93 |  |  |  |  |  |
| Ni(NO_3_)_2_·6H_2_O | $139.98 |  |  |  |  |  |
| Chitosan | $76.05 |  |  |  |  |  |
| Extra C_E_ & C_M_ | $83.76 (20%) |  |  |  |  |  |
| **Graphite flakes** | $0.39 | **1256.91** | **1.394** | **0.1** | **120** | **46** |
| H_2_SO_4_, H_3_PO_4_ | $8.91 |  |  |  |  |  |
| KMnO_4_ | $23.67 |  |  |  |  |  |
| HAuCl_4_·3H_2_O | $939.3 |  |  |  |  |  |
| Sodium citrate | $0.19 |  |  |  |  |  |
| Balsa wood | $33.07 |  |  |  |  |  |
| Extra C_E_ & C_M_ | $251.38 (20%) |  |  |  |  |  |
| **Rice husk** | $14.48 | **907.73** | **1.71** | **0.2** | **84** | **47** |
| Acrylamide | $3.62 |  |  |  |  |  |
| N,N’-methylenebisacrylamide | $198.22 |  |  |  |  |  |
| Sodium dodecyl sulfate | $463.68 |  |  |  |  |  |
| N,N,N’,N’tetramethylethylenediamin | $0.32 |  |  |  |  |  |
| Na_2_S_2_O_8_ | $48.29 |  |  |  |  |  |
| Na_2_SO_3_ | $7.39 |  |  |  |  |  |
| Extra C_E_ & C_M_ | $90.77(10%) |  |  |  |  |  |
| **Raw material prices and cost of equipment (C_E_) and manpower (C_M_)** | **Estimated single item cost ($/1*1*0.01 m^-3^)** | **Total cost ($/1*1*0.01 m^-3^)** | **Evaporation rate (kg m^-2^ h^-1^)** | **Cost-effectiveness (kg h^-1^ $^-1^)** | **Durability (h)** | **Ref.** |
| **Maize straw** | $0.76 | **210.77** | **2.71** | **1.3** | **4** | **48** |
| Graphite flakes | $0.06 |  |  |  |  |  |
| H_2_SO_4_, H_3_PO_4_ | $53.04 |  |  |  |  |  |
| KMnO4 | $144.69 |  |  |  |  |  |
| Na_2_SO_3_ | $9.94 |  |  |  |  |  |
| Sodium dodecyl sulfate | $0.1 |  |  |  |  |  |
| Ascorbic acid | $2.18 |  |  |  |  |  |
| Extra C_E_ & C_M_ | $21.07 (10%) |  |  |  |  |  |
| **Pomelo peel** | $11.47 | **12.04** | **1.39** | **11.5** | **84** | **49** |
| Extra C_E_ & C_M_ | $0.57 (5%) |  |  |  |  |  |
| **Tannic acid** | $226.33 | **339.69** | **1.83** | **0.5** | **35** | **50** |
| FeCl_3_·6H_2_O | $8.45 |  |  |  |  |  |
| Coconut husk | $70.94 |  |  |  |  |  |
| Extra C_E_ & C_M_ | $33.97 (10%) |  |  |  |  |  |
| **Copper foam** | **$38** | **61.37** | **1.71** | **2.8** | **90** | **Our work** |
| **NaOH (NH_4_)_2_S_2_O_8_** | **$1.2** |  |  |  |  |  |
| **Graphene oxide** | **$10.1** |  |  |  |  |  |
| **Sodium polystyrene sulfonate** | **$6.55** |  |  |  |  |  |
| **Air-laid paper** | **$0.02** |  |  |  |  |  |
| **Extra C_E_ & C_M_** | **$5.5 (10%)** |  |  |  |  |  |

**SI-19. Performance Comparison**

We compared the evaporation rate and durability of salt discharge evaporators reported in recent years using a two-phase area plot. Although our reported evaporation rate is not the most advanced level, the duration is still at a considerable excellent level. We strive to improve the operational lifespan and stability of the evaporator without sacrificing its evaporation rate, achieving a balance between high evaporation rate and high sustainability.

**
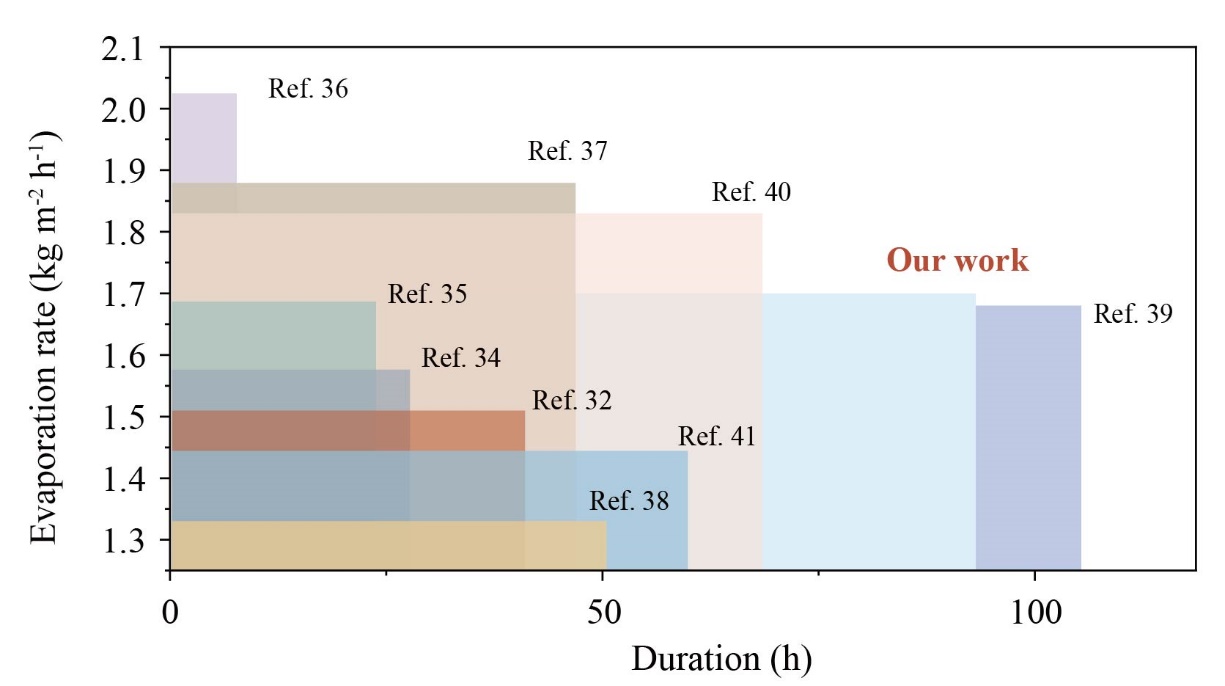
**

**Figure S13 Comparison chart of pure evaporation rate (kg m^-2^ h^-1^) and durability or long-term duration tests (hours) for different evaporators from the literature.**

**SI-20. SI Video**

Video SI1-SHiCF-GO-PSS-CapillaryRaise shows the rising process of the wetting front in the case of direct contact between the SHiCF-GO-PSS evaporator and bulk seawater. Water is pumped up from the bottom of the SHiCF-GO-PSS interface through capillary action and completely diffused, resulting in filling state (light reflection) of the bottom part of the evaporator, water film wetting state of the middle part of evaporator and dry state of the upper part of the evaporator.

**References**

[1] F. Lv, J. Miao, J. Hu, D. Orejon, *Small* **2023**, 19, 2208137.

[2] P. Liu, Y. B. Hu, X. Y. Li, L. Xu, C. Chen, B. Yuan, M. L. Fu, *Angew Chem Int Ed Engl* **2022**, 61, e202208587.

[3] X. Wang, Q. Gan, R. Chen, H. Peng, T. Zhang, M. Ye, *ACS Sustainable Chemistry & Engineering* **2020**, 8, 7753.

[4] W. Zhao, H. Gong, Y. Song, B. Li, N. Xu, X. Min, G. Liu, B. Zhu, L. Zhou, X. X. Zhang, J. Zhu, *Advanced Functional Materials* **2021**, 31, 2100025.

[5] H. Zhang, Y. Du, D. Jing, L. Yang, J. Ji, X. Li, *ACS Appl Mater Interfaces* **2023**, 15, 49892.

[6] P. A. Obraztsov, M. G. Rybin, A. V. Tyurnina, S. V. Garnov, E. D. Obraztsova, A. N. Obraztsov, Y. P. Svirko, *Nano Lett* **2011**, 11, 1540.

[7] C. Chen, Y. Kuang, L. Hu, *Joule* **2019**, 3, 683.

[8] N. Cao, S. Lu, R. Yao, C. Liu, Q. Xiong, W. Qin, X. Wu, *Chemical Engineering Journal* **2020**, 397, 125522.

[9] Z. Yang, D. Li, K. Yang, L. Chen, J. Wang, X. Zhu, B. Chen, *Environ Sci Technol* **2023**, 57, 13047.

[10] a) Y. Wang, C. Wang, X. Song, M. Huang, S. K. Megarajan, S. F. Shaukat, H. Jiang, *Journal of Materials Chemistry A* **2018**, 6, 9874; b) L. Li, N. He, B. Jiang, K. Yu, Q. Zhang, H. Zhang, D. Tang, Y. Song, *Advanced Functional Materials* **2021**, 31, 2104380.

[11] C. Tian, J. Liu, R. Ruan, X. Tian, X. Lai, L. Xing, Y. Su, W. Huang, Y. Cao, J. Tu, *Small* **2020**, 16, 2000573.

[12] Y. Yang, R. Zhao, T. Zhang, K. Zhao, P. Xiao, Y. Ma, P. M. Ajayan, G. Shi, Y. Chen, *ACS Nano* **2018**, 12, 829.

[13] J. Miao, F. Lv, R. Gulfam, W. Zhao, *Applied Energy* **2023**, 350, 121779.

[14] M. K. Alam, M. He, W. Chen, L. Wang, X. Li, X. Qin, *ACS Appl Mater Interfaces* **2022**, 14, 41114.

[15] J. Chen, J. L. Yin, B. Li, Z. Ye, D. Liu, D. Ding, F. Qian, N. V. Myung, Q. Zhang, Y. Yin, *ACS Nano* **2020**, 14, 17419.

[16] D. Deng, Q. Liang, Z. Xiao, C. Liu, *Chemical Engineering Journal* **2023**, 474, 145422.

[17] X. Huang, L. Li, X. Zhao, J. Zhang, *J Colloid Interface Sci* **2023**, 646, 141.

[18] Y. Li, X. Wang, R. Wu, J. Qin, Y. Fu, M. Qin, Y. Zhang, C. Xu, *Industrial Crops and Products* **2023**, 206, 117649.

[19] P. Qiao, J. Wu, H. Li, Y. Xu, L. Ren, K. Lin, W. Zhou, *ACS Appl. Mater. Interfaces* 2019, 11, 7066–7073.

[20] J. Chen, J. Feng, Z. Li, P. Xu, X. Wang, W. Yin, M. Wang, X. Ge, Y. Yin, *Nano Lett.* 2018, 19, 400–407.

[21] K. Yin, S. Yang, J. Wu, Y. Li, D. Chu, J. He, J.-A. Duan, *J. Mater. Chem. A,* 2019, 7, 8361–8367.

[22] X. Li, J. Li, J. Lu, N. Xu, C. Chen, X. Min, B. Zhu, H. Li, L. Zhou, S. Zhu, *Joule*, 2018, 2, 1331–1338.

[23] L. Cui, P. Zhang, Y. Xiao, Y. Liang, H. Liang, Z. Cheng, L. Qu, *Adv. Mater.*, 2018, 30, 1706805.

[24] Y. Xu, H. Xu, Z. Zhu, H. Hou, J. Zuo, F. Cui, D. Liu, W. Wang, *J. Mater. Chem. A* 2019, 7, 22296–22306.

[25] Z. Sun, J. Wang, Q. Wu, Z. Wang, Z. Wang, J. Sun, C. J. Liu, *Adv. Funct. Mater.*, 2019, 29, 1901312.

[26] Y. Geng, K. Zhang, K. Yang, P. Ying, L. Hu, J. Ding, J. Xue, W. Sun, K. Sun, M. Li, *Carbon* 2019, 155,25–33.

[27] X. Li, R. Lin, G. Ni, N. Xu, X. Hu, B. Zhu, G. Lv, J. Li, S. Zhu, J. Zhu, *Natl. Sci. Rev.*, 2017, 5,70–77.

[28] X. Wang, Q. Liu, S. Wu, B. Xu, H. Xu, *Adv. Mater.*, 2019, 31, 1807716.

[29] Y. Guo, X. Zhou, F. Zhao, J. Bae, B. Rosenberger, G. Yu, *ACS Nano*, 2019, 13, 7913–7919.

[30] F. Zhao, X. Zhou, Y. Shi, X. Qian, M. Alexander, X. Zhao, S. Mendez, R. Yang, L. Qu, G. Yu, *Nat. Nanotechnol.*, 2018, 13, 489–495.

[31] Z. Liu, R. Qing, A. Xie, H. Liu, L. Zhu, S. Chen, *ACS Appl. Mater. Interfaces* 2021, 13, 18829−18837.

[32] X. Sun, X. Jia, J. Yang, S. Wang, Y. Li, D. Shao, H. Song, *J. Mater. Chem. A* 2021, 9, 23891.

[33] J. Yang, Y. Chen, X. Jia, Y. Li, S. Wang, H. Song, *ACS Appl. Mater. Interfaces* 2020, 12, 47029.

[34] Z. Wu, K. Li, R. Deng, Y.-R. Xue, H.-N. Li, H.-C. Yang, Z.-K. Xu, *Chem. Eng. J.* 2024, 500, 156851.

[35] X. Dong, L. Cao, Y. Si, B. Ding, H. Deng, *Adv. Mater.* 2020, 32, 1908269.

[36] C. Gao, J. Zhu, J. Li, B. Zhou, X. Liu, Y. Chen, Z. Zhang, J. Guo, J. *Colloid Interface Sci.* 2022, 619, 322.

[37] Z. Wang, C. Wu, X. Wang, M. Xie, Y. Li, Z. Zhan, Y. Shuai, *Adv. Funct.. Mater.* 2024, 2416014. https://doi.org/10.1002/adfm.202416014

[38] C. Shi, Z. Wu, Y. Li, X. Zhang, Y. Xu, A. Chen, C. Yan, Y. Shi, T. Wang, B. Su, *ACS Appl. Mater. Interfaces* 2023, 15, 23971.

[39] F. Wang, C. Wang, G. Shi, Y. Wang, F. Li, K. Xu, M. Ma, *Desalination* 2023, 545, 116157.

[40] X. Wang, L. Zhang, D. Zheng, X. Xu, B. Bai, M. Du, *Chem. Eng. J.* 2023, 462, 142265.

[41] X. Hao, H. Yao, P. Zhang, Q. Liao, K. Zhu, J. Chang, H. Cheng, J. Yuan, L. Qu, *Nat. Water* 2023, 1, 982.

[42] M. Zhu, Y. Li, F. Chen, X. Zhu, J. Dai, Y. Li, Z. Yang, X. Yan, J. Song, Y. Wang, E. Hitz, W. Luo, M. Lu, B. Yang, L. Hu, *Advanced Energy Materials* 2017, 8, 1701028.

[43] J. Wu, Z. Cui, Y. Yu, B. Yue, J. Hu, J. Qu, J. Li, D. Tian, Y. Cai, Advanced. Science 2023, 10, 2305523.

[44] F. Yu, Z. Chen, Z. Guo, M. S. Irshad, L. Yu, J. W. Qian, T. Mei and X. B. Wang, *ACS Sustain. Chem. Eng.*, 2020, 8, 7139−7149.

[45] P. F. Wang, X. Y. Wang, S.Y. Chen, J. H. Zhang, X. J. Mu, Y. L. Chen, Z. Q. Sun, A. Y. Wei, Y. Z. Tian, J. H. Zhou, X. X. Liang and L. Miao, N. Saito, *ACS Appl. Mater. Interfaces*, 2021, 13, 30556−30564.

[46] Y. Chen, J. Yang, L. Zhu, X. H. Jia, S. Z. Wang, Y. Li and H. J. Song, *J. Mater. Chem. A*, 2021, 9, 15482-15492.

[47] P. P. He, L. Hao, N. Liu, H. Y. Bai, R. Niu and J. Gong, Chem. Eng. J., 2021, 423, 130268.

[48] Q. Zhang, L. Li, B. Jiang, H. T. Zhang, N. He, S. Yang, D. W. Tang and Y. C. Song, *ACS Appl. Mater. Interfaces*, 2020, 12, 28179−28187.

[49] X. X. Chen, Z. Y. Wu, D. G. Lai, M. Zheng, L. Xu, J. B. Huo, Z. X. Chen, B. L. Yuan and M. L. Fu, *J. Mater*. *Chem. A*, 2020, 8, 22645-22656.

[50] Y. Kong, H. B. Dan, W. J. Kong, Y. Gao, Y. N. Shang, K. D. Ji, Q. Y. Yue and B. Y. Gao, *J. Mater. Chem. A*, 2020, 8, 24734-24742.

[51] X. H. Liu, D. D. Mishra, Y. K. Li, L. Gao, H. Y. Peng, L. Zhang and C. Q. Hu, ACS Sustain. *Chem. Eng.*, 2021, 9, 4571−4582.

[52] T. T. Phama, T. H. Nguyenb, T. A. H. Nguyena, D. D. Phama, D. C. Nguyenc, D. B. Do, H. V. Nguyend, M. H. Ha and Z. H. Nguyen, *Desalination*, 2021, 518, 115280.

1. * Corresponding author. Email address: [fengyonglv@alumni.sjtu.edu.cn](mailto:fengyonglv@alumni.sjtu.edu.cn) (F. Y. Lv); † Corresponding author. Email address: hujing616@126.com (J. Hu); ‡ Corresponding author. Email address: D.Orejon@ed.ac.uk (D. Orejon) [↑](#footnote-ref-1)
